# Clarithromycin inhibits autophagy in colorectal cancer by regulating the hERG1 potassium channel interaction with PI3K

**DOI:** 10.1038/s41419-020-2349-8

**Published:** 2020-03-02

**Authors:** Giulia Petroni, Giacomo Bagni, Jessica Iorio, Claudia Duranti, Tiziano Lottini, Matteo Stefanini, Goran Kragol, Andrea Becchetti, Annarosa Arcangeli

**Affiliations:** 1 Department of Experimental and Clinical Medicine, University of Firenze, Viale GB Morgagni 50, 50134 Firenze, Italy; 2 Fidelta Ltd., Prilaz baruna Filipovića 29, 10000 Zagreb, Croatia; 3 Department of Biotechnology and Biosciences, University of Milano-Bicocca, Piazza della Scienza 2, 20126 Milano, Italy

**Keywords:** Gastrointestinal cancer, Autophagy, Cell death

## Abstract

We have studied how the macrolide antibiotic Clarithromycin (Cla) regulates autophagy, which sustains cell survival and resistance to chemotherapy in cancer. We found Cla to inhibit the growth of human colorectal cancer (CRC) cells, by modulating the autophagic flux and triggering apoptosis. The accumulation of cytosolic autophagosomes accompanied by the modulation of autophagic markers LC3-II and p62/SQSTM1, points to autophagy exhaustion. Because Cla is known to bind human Ether-à-go-go Related Gene 1 (hERG1) K^+^ channels, we studied if its effects depended on hERG1 and its conformational states. By availing of hERG1 mutants with different gating properties, we found that fluorescently labelled Cla preferentially bound to the closed channels. Furthermore, by sequestering the channel in the closed conformation, Cla inhibited the formation of a macromolecular complex between hERG1 and the p85 subunit of PI3K. This strongly reduced Akt phosphorylation, and stimulated the p53-dependent cell apoptosis, as witnessed by late caspase activation. Finally, Cla enhanced the cytotoxic effect of 5-fluorouracil (5-FU), the main chemotherapeutic agent in CRC, in vitro and in a xenograft CRC model. We conclude that Cla affects the autophagic flux by impairing the signaling pathway linking hERG1 and PI3K. Combining Cla with 5-FU might be a novel therapeutic option in CRC.

## Introduction

Autophagy is a homeostatic and evolutionarily conserved process characterized by cellular self-digestion and the removal of defective organelles and proteins, aimed to maintain cellular biosynthesis during nutrient deprivation or metabolic stress^1^. The autophagic signaling pathways are altered in a variety of diseases^2^. In cancer, autophagy serves apparently conflicting roles, as it can regulate tumor suppressing as well as promoting activities, depending on the cellular context and tumor stage. During early tumorigenesis, autophagy acts as a protective mechanism against malignant transformation. As the tumor progresses, however, autophagy can be stimulated to allow cancer cells to survive microenvironmental stress and to increase aggressiveness and drug resistance^2,3^. Nonetheless, in certain circumstances, autophagy constitutes a major mechanism for cell killing, or can activate other death pathways^4^.

Such dual role of autophagy is clearly observed in colorectal cancer (CRC), one of the most aggressive cancer types. Indeed, many genes and proteins involved in the autophagic process are implicated in CRC tumor progression^5–9^. However, the role of autophagy in progression and survival of CRC patients is still controversial^7,10–14^.

Targeting autophagy represents a promising strategy also for CRC therapy^5,6^. A number of in vitro^15–19^ and in vivo^19–22^ preclinical studies have shown that inhibiting autophagy enhances CRC cell death and could be used to restore chemosensitivity. In this context, macrolide antibiotics, such as clarithromycin (Cla), erythromycin (Er), and azithromycin, have been proven to exert antitumor activity in several preclinical models of cancer, including CRC, by modulating the autophagic flux^23–31^. Macrolide antibiotics were effective either alone or in combination with conventional treatments^24–27,29,31^. Besides inhibiting the late phase of autophagy, macrolide antibiotics can exert their antitumor effects through different mechanisms, e.g., by inducing apoptosis^30–32^, inhibiting tumor-induced angiogenesis^33^, or acting as a chemopreventive agent^34^.

Despite the wide pharmacological evidence, virtually nothing is known about the signaling pathways by which macrolides exert their cellular effects. Presently, the only identified Cla targets in mammalian cell membranes are the K^+^ channel encoded by the *ether-à-go-go related gene 1* (*hERG1*, also known as *Kv11.1*)^35,36^, and the solute carrier organic anion transporters *SLCOB1* and *SLCOB3*^37^. hERG1 is often aberrantly expressed in cancers^38–41^, including CRC^42^, and we have previously shown that hERG1 operates in a peculiar way in cancer cells, mainly modulating the intracellular signaling triggered by cell adhesion^43^. In particular, in CRC cells, hERG1 forms a signaling complex with the p85 subunit of PI3K, which activates the Akt/HIF(s) pathway^44^.

In this paper, we investigated how the interplay between hERG1 and the PI3K/Akt pathway is regulated by Cla, and how this mechanism regulates the autophagic effects of the macrolide. Moreover, we examined whether the effects of Cla on autophagy could sensitize human CRC cells to chemotherapeutics agents, both in vitro and in vivo.

## Materials and methods

### Patients and sample collection

A retrospective study was conducted on a cohort of 127 patients with colorectal adenocarcinoma, selected by the medical oncologists of Azienda Ospedaliero Universitaria, Careggi, Florence and of the Spedali Civili Hospital, Brescia. Healthy mucosa (*n* = 13), stage I (*n* = 20), II (*n* = 34), III (*n* = 42), and IV (*n* = 18) patients were treated with surgery in both institutions. The study was carried out with approval of the local Ethical Committee (BIO.14.033). The local Ethical Committee decided the sample size. All the patients were enrolled after informed written consent. The study was performed in accordance with the Declaration of Helsinki.

### Immunohistochemistry (IHC) analysis

IHC was performed on formalin-fixed, paraffin-embedded samples belonging to either CRC patients or HCT116 tumor masses obtained after xenografting in mice. IHC were carried out as previously reported^45,46^ using the antibodies listed in Table S1, and applying a commercially available kit (PicTure max kit; Invitrogen) according to the manufacturer’s instructions. Samples were evaluated by three independent investigators.

### Cell cultures and treatment with clarithromycin

HCT116 and LS174T, and HEK293 cells were obtained from the American Type Culture Collection (ATCC); HT29 cells and HCT116 p53^−/−^ were kindly provided by Dr. R. Falcioni (Regina Elena Cancer Institute, Roma). Cells were routinely cultured at 37 °C with 5% CO_2_ in a humidified atmosphere, in RPMI (Euroclone) (HCT116, HT29, and HCT116 p53^−/−^), ATCC-formulated Eagle’s Minimum Essential Medium (EMEM; ATCC) (LS174T) or Dulbecco’s modified Eagle’s Medium (DMEM; Euroclone) (HEK293), supplemented with 2% l-Glut and 10% fetal bovine serum (Euroclone). We certify that all the cell lines used in the paper were screened for Mycoplasma contamination. Only Mycoplasma negative cells were used in the present study. HCT116 and HEK293 cells expressing wild type (WT) or mutant hERG1 constructs were prepared as previously described^47,48^. Selection and subsequent cell culture maintenance were performed in complete culture medium supplemented with either 1.6 mg/ml (for HCT116 cells) or 0.8 mg/ml (for HEK293 cells) Geneticin (G418, Invitrogen). Silencing of *hERG1* in HCT116 cells, was carried out with siRNAs as previously described^44^. For treatment with Cla and the other drugs, cells were seeded at the following concentrations: 1 × 10^4^ cells/well in 96 wells-plate for cytotoxic assays; 5 × 10^4^ cells/well in 24 wells-plate for evaluating autophagy and apoptosis by flow cytometry; 5 × 10^5^ cells/well in 6 wells-plate for protein extraction. After overnight incubation in complete medium, the medium was changed and cells were incubated for different times in control conditions (complete medium plus the vehicle) and in medium containing Cla or the other drugs.

### Chemicals

Unless otherwise indicated, chemicals, drugs and antibodies were from Sigma-Aldrich. The details of the use for either in vitro or in vivo experiments are given in Table S2. All stock solutions were stored at −20 °C.

### Cell viability assay

Cell viability was measured by the Trypan Blue exclusion test. After incubation with the drugs, the Trypan Blue dye was added to the harvested cells and live cells counted with a hemocytometer. The 50% inhibitory concentration (IC_50_) and combination index (CI) calculation were performed as previously described^49^.

### Evaluation of autophagic vacuoles

After treatment, cells were harvested and cytospun onto glass slides, and stained with May-Grünwald and Giemsa, as previously described^30^. Vacuoles’ diameter was calculated with ImageJ (ImageJ 1.38, U.S. National Institutes of Health). Acridine orange (AO) staining was performed staining treated cells with AO (1 μm/mL) in complete medium for 15 min at 37 °C. The staining was evaluated with a fluorescence microscope Nikon Eclipse TE300 and by flow cytometry. Data were analyzed through the BD FACSDiva Software 6.1.3.

### Flow cytometry

Cell cycle distribution was assessed by flow cytometry after staining the cells with propidium iodide (PI) as previously described^50^. The percentage of apoptotic cells was determined using the Annexin-V/PI test (Annexin-V FLUOS staining kit; Roche Diagnostics, Mannheim, Germany) as previously described^50^. The generic caspase activity assay kit (Fluorometric-Green; cat. no. ab112130; Abcam, Cambridge, UK) was used to detect the activity of caspases 1–9, as previously described^51^.

### Western blot (WB) and co-immunoprecipitation (co-IP)

Protein lysates and WBs relative to cell lines and tumor masses were performed as previously described^44^. For the co-IP of hERG1 and the p85 subunit of PI3K, the procedure described in ref. ^44^ was followed. To quantify variations in hERG1–p85 interactions, the signal for the co-immunoprecipitated protein (p85) was first divided by the signal of the protein used for immunoprecipitation (hERG1) and then normalized to the signal of the corresponding protein in the total lysate (input hERG1). The resulting value is indicated as “p85/hERG1 complex”. The list of antibodies and the concentration used for WBs are in Table S1. WB images were acquired with an Epson 3200 scanner. Densitometric analysis was performed using ImageJ on two different scans, after background subtraction, from at least three different experiments, as described in ref. ^48^.

### Cla-binding assay

Cla binding to hERG1 was assessed by using fluorescently labeled 11-O-{3-[(7-nitro-2,1,3-benzoxadiazol-4-yl)amino]propyl}-6-O-methyl-erythromycin A (shortly: 11-NBD-Cla), synthesized as reported^52^, on normal human embryonic kidney (HEK)293 cells transfected with hERG1 and different hERG1 mutants. Cells were seeded in 96-wells black assay plates (Corning Incorporated, Kennebunk, ME, USA) at 1 × 10^4^ cells/well in complete medium. After 24 h, cells were treated for 30 min with 10 µM 11-NBD-Cla at 37 °C. After a brief wash at room temperature with phosphate-buffered saline (PBS), fluorescence intensity was immediately measured with a Synergy H1 microplate reader (BioTek Instruments, Winooski, VT, USA) (excitation/emission 463/536 nm). Cells were then lysed in 0.5% Triton X-100 for 15 min on ice and protein concentration was determined by Bio-Rad protein assay (Bio Rad, Hercules, USA). Fluorescence intensity was normalized on total protein content, after subtracting the values obtained from HEK293 MOCK cells. The obtained data were normalized on the relative hERG1 expression in HEK293 cells transfected with the different mutants, shown in ref. ^48^. Obtained results are hence referred to as “11-NBD-Cla fluorescence increase relative to MOCK cells” in Fig. 3e.

### Immunofluorescence (IF) and confocal imaging

IF experiments were performed with the antibody reported in Table S1, using an overnight incubation. Briefly, cells seeded onto glass slides and incubated with Cla, were fixed in 4% paraformaldehyde in PBS and permeabilized with 1% Triton X-100 in PBS. Anti-rabbit-Cy2 (1:1000) was used as secondary antibody, and nuclei were counterstained with Hoechst 33342 dye. Slides were examined with a Nikon Eclipse TE2000-U confocal microscope (Nikon, Tokyo, Japan).

To assess 11-NDB Cla binding by IF, HEK293 transfected with the different hERG1mutants were seeded on glass coverslips in 24-wells plates at a density of 3 × 10^4^ cells/well in complete medium. After 24 h, cells were treated for 30 min with 10 µM 11-NBD-clarithromycin, washed once with PBS at room temperature and fixed in 4% PFA for 15 min. Slides were imaged using a Nikon Eclipse TE2000-U confocal microscope (Nikon, Tokyo, Japan) (z-stacks steps = 0.5 µm, laser detection for 11-NBD-Cla = 450/35–515/30). Two types of analysis were then performed on exported confocal images: (i) mean 11-NBD-Cla fluorescence intensity was measured at the top focal plane of the image z-stack and normalized on selected cell areas using Fiji^53^ and (ii) mean fluorescence intensity was also quantified in single plane 2D confocal images and normalized on selected cell area using ImageJ.

### 3D cell model

Multicellular tumor spheroids were formed seeding 10^3^ HCT116 cells per well, on an agarose-coated 96-well flat-bottom plate. After 72 h, the homogeneity of spheroid size was checked for each well and spheroids were treated with drugs. Spheroid volumes were automatically calculated by ImageJ. Drug induced death was evaluated by Calcein-AM/PI staining All the procedures were as in ref. ^54^.

### In vivo tumor xenograft models

Experiments were performed at L.I.Ge.M.A. (Laboratory of genetic engineering for the production of mouse model) in the Animal House of the University of Florence (CESAL). Mice were housed in filter-top cages with a 12-h dark–light cycle, and had unlimited access to food and water. Procedures were conducted according to the laws for experiments on live animals (Directive 2010/63/EU), and approved by the Italian Ministry of Health (619/2016-PR). The study was carried out with approval of the local Animal Welfare Committee (AWC). The animal sample size (α error prob: 0.05; Power: 0,8) was determined using the G*Power software and approved by the local AWE.

Female nude mice Foxn1^nu^ mice (Envigo Laboratories) aged 5–6 weeks were injected subcutaneously in either flank with 3 × 10^6^ HCT116 cells, as in ref. ^55^. After cell inoculation, mice were monitored daily to ensure they did not show any signs of suffering or disease (such as weight loss, abdominal distension, impaired movement, and edema in the injection area). One week after inoculation, mice were treated for 2 weeks with saline as reported in Table S2 and in the schematic representation of treatment regime in Fig. 7f. Mice were randomized before the treatment, using with a single sequence of random assignments (simple randomization) with the GraphPad software. During the treatments mice health was monitored constantly, in order to discard from the study all mice with signs of suffering.

Three weeks after inoculation, mice were sacrificed, and tumor masses were collected. Tumor growth was monitored by in vivo Ultrasound imaging and 3D scans of the tumors were performed in B-Mode imaging with VevoLAZR-X (FUJIFILM VisualSonics). The tumor areas were measured delineating the margins (region of interest, ROI) for every axial slide using Vevo LAB software. During ultrasound imaging mice were anesthetized by 1.5/2% isoflurane and placed on a pad heated at 37 °C.

### Statistical analysis

Unless otherwise indicated, data were obtained from at least three independent experiments and are given as mean values ± standard error of the mean (SEM). Statistical comparisons were performed with OriginPro 8 (Origin Lab, Northampton, Massachusetts), when we had a least three independent experiments. The normality of data distribution was checked with Kolmogorov–Smirnov test, and the variance homogeneity with *F*-test. In the case of normal distributions, each data set was checked for variance homogeneity, using the *F*-test for equality of two variances and the Brown–Forsythe test for multiple comparisons.

For comparisons between two groups, we used Student’s *t* test. In case of multiple comparisons, one-way ANOVA followed by Bonferroni’s post hoc test was performed. All the data reported meet the assumptions of the tests. Test of normality distribution and variance homogeneity assumptions have been proper performed and used to choose the right test for compare groups.

For data reported in the paper as “normalized” or “fold change”, statistical analysis has been performed on original (non-normalized) data.

Statistical tests are proper performed and used correctly to compare groups for every figure.

## Results

### Clarithromycin affects autophagy of human CRC cells

**Fig. 1:**
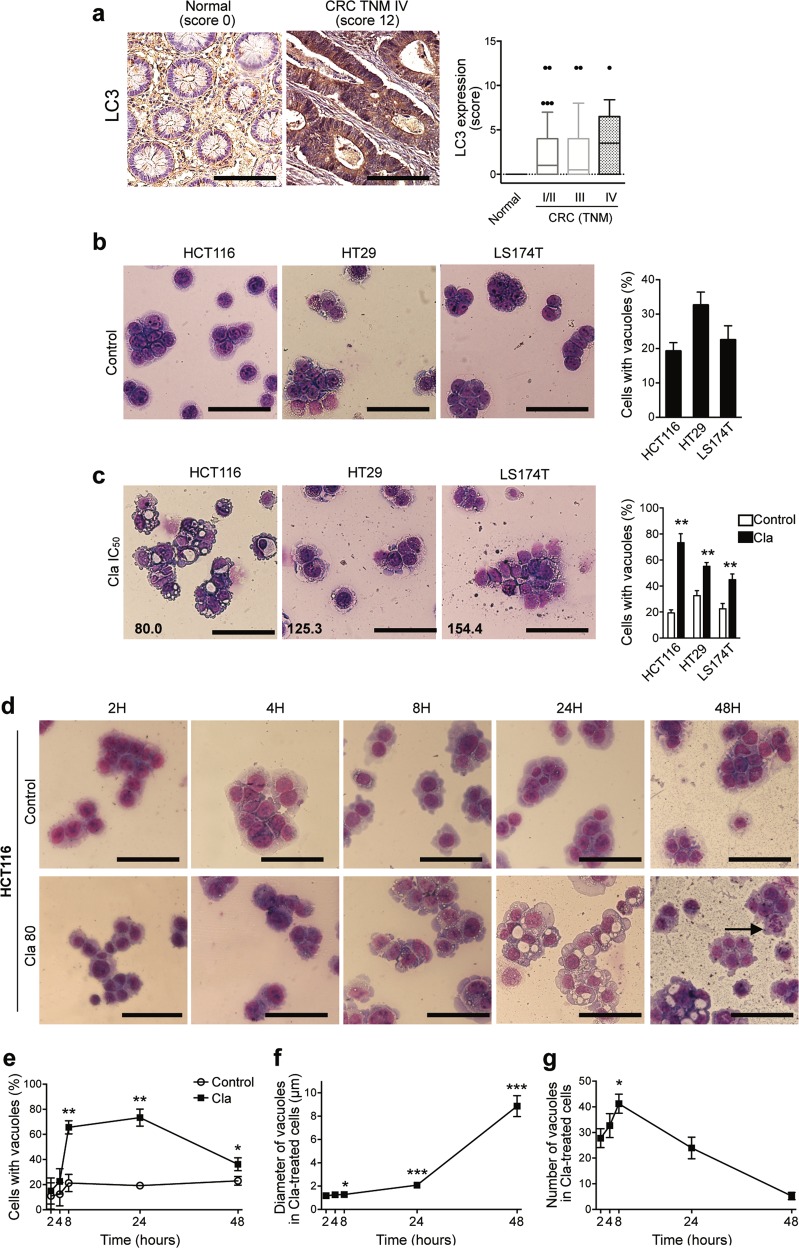
Human CRC displays high basal autophagy that is affected by Clarithromycin treatment. **a** Human normal colorectal (*n* = 13) and CRC (*n* = 114) tissues were sectioned and subjected to IHC staining against LC3. Representative images of a normal colorectal and of a CRC specimen with a high score (original magnification, ×400; scale bar 100 μm) are reported. LC3 staining was evaluated as previously described, multiplying the percentage of immunoreactive cells (quantity score) with the estimate of staining intensity (staining intensity score)^46^. No staining was scored as 0, 1–25% of stained cells was scored as 1, 26–50% 30 as 2, 51–75% as 3, and 76–100% as 4. Staining intensity was rated on a scale of 0–3, with 0 = negative; 1 = weak; 2 = moderate, and 3 = strong. LC3 expression (score), is reported in the graph for normal and CRC patients stratified according to the tumor/node/metastasis (TNM) system. **b**, **c** HCT116, HT29, and LS174T cells were incubated for 24 h in complete medium (**b**) or treated with Cla (**c**) at their relative IC_50_ values (indicated in the lower left corner of each picture), then stained with May–Grümwald and Giemsa in order to evaluate vacuoles formation. Percentages of cells with vacuoles are reported in the bar graphs as mean ± SEM (*n* = 3). **d**–**g** Time course (2–48 h) of vacuoles formation in HCT116 cells treated with Cla (80 µM). Representative images of HCT116 cells stained with May-Grümwald and Giemsa, are reported in (**d**). The arrow shows an early apoptotic cell with nuclear fragmentation. **e** Percentages of cells with vacuoles, relative to each time point. The effects of Cla on size (**f**) and number (**g**) of vacuoles were quantified by measuring the diameters of all vacuoles and counting all vacuoles in 10–12 cells per each time point, respectively, from three independent experiments. For **f** and **g** only HCT116 cells treated with Cla which presented visible vacuoles were evaluated. **b**–**d** Representative images of three independent experiments are reported (original magnification ×400; scale bar 100 µm). Statistical significance was assessed with a one-way ANOVA for (**c**, **e**, **f**, **g**); *P < 0.05; **P < 0.01, and ***P < 0.001.

Autophagy was previously observed in CRC^7–14^. We confirmed this in 127 primary human CRC surgical samples, by immunohistochemical labeling of the microtubule-associated protein 1 light chain 3 (LC3). LC3 was absent in normal colonic human mucosa, but was highly expressed in cancer specimens (Fig. 1a). The percentage of cases showing a positive score (≥1) was approximately 60%, irrespective of the tumor/node/metastasis (TNM) stage, whereas the labeling score (assessed as in ref. ^46^), slightly increased from TNM stages I–III to TNM stage IV samples (Fig. 1a). A significant autophagy, determined as the percentage of cells with intracytoplasmic vacuoles was also detected in three human CRC cell lines, HCT116, HT29, and LS174T, in basal culture conditions (Fig. 1b).

The same human CRC cell lines were studied to test the effects of Cla on autophagy as above. The drug was applied at the IC_50_ value, based on the results given in Fig. S1 and Table 1a. Cla induced the formation of numerous intracytoplasmic vacuoles after 24 h, in all cell lines, especially in HCT116 cells (Fig. 1c), which were hence used as a model. The percentage of HCT116 cells with vacuoles significantly increased after 8 h of treatment, up to 24 h (Fig. 1d, e). Subsequently, the size of vacuoles increased (Fig. 1d, f), whereas their number per cell decreased up to 48 h of incubation (Fig. 1d, g). At this time, cells began to display nuclear fragmentation, a typical feature of apoptotic cells (Fig. 1d). The decrease in number and the increase in size of intracytoplasmic vacuoles were more evident when a dose equal to 2 × IC_50_ (i.e., 160 μM) was used (Fig. S2). Similar effects were detected in all the human CRC cell lines we tested (Fig. S2).

**Table 1:** Fifty percent inhibitory concentration (IC_50_) and combination index (CI) of Clarithromycin, Irinotecan, 5-Fluorouracil, Cisplatin, and Oxaliplatin for the reported cancer cell lines.

(a) IC_50_ values of Clarithromycin for different cell lines				
**Drug**	**Cell line**		**IC**_**50**_ **(μM)**	
*Clarithromycin*				
	HCT116		80.0 ± 2.0	
	HT29		125.3 ± 16.8	
	LS174T		154.4 ± 9.1	
	HCT116 p53^−/−^		150.0 ± 8.6	
	HEK293		>200	
	HEK293-MOCK		>200	
	HEK293-hERG1		92.4 ± 4.9	
**(b) IC**_**50**_ **and IC**_**25**_ **values of Irinotecan (CPT11), 5-Fluorouracil (5-FU), Cisplatin (Cis), and Oxaliplatin (Oxa), and their combination index (CI) with Clarithromycin for HCT116 cells**				
**Drug**	**IC**_**50**_ **(μM)**	**IC**_**25**_ **(μM)**	**CI at IC**_**50**_ **(effect)**	**CI at IC**_**25**_ **(effect)**
CPT-11	10.1 ± 2.1	2.1 ± 0.1	0.63 ± 0.09 (S)	0.41 ± 0.02 (S)
5-FU	13.7 ± 2.4	2.21 ± 0.1	0.57 ± 0.07 (S)	0.38 ± 0.03 (S)
Cis	25.2 ± 2.1 (Ref. ^50^)	9.04 ± 0.7	1.38 ± 0.06 (A)	0.87 ± 0.15 (S)
Oxa	57.4 ± 9.4 (Ref. ^50^)	12.2 ± 3.0	1.10 ± 0.11 (A)	0.78 ± 0.10 (S)
**(c) IC**_**50**_ **and IC**_**25**_ **values of Clarithromycin and 5-Fluorouracil for HCT116 cells cultured as spheroids**				
**Drug**	**IC**_**50**_ **(μM)**		**IC**_**25**_ **(μM)**	
Clarithromycin	>300		191.8 ± 21.3	
5-FU	260.8 ± 4.6		41.7 ± 8.9	

(a) IC_50_ values were determined after 24 h of treatment by the Trypan Blue exclusion test, using Origin Software. Data are means ± SEM of four independent experiments, each carried out in triplicate. (b) HCT116 cells were exposed to Irinotecan (CPT-11), 5-Fluorouracil (5-FU), Cisplatin (Cis) or Oxaliplatin (Oxa) alone or in combination with Cla for 24 h as described in ref. ^50^. IC_50_ and IC_25_ vales were determined as in (a). For the determination of the CI the drugs were used at their IC_50_ and IC_25_ values. CI values were calculated using Calcusyn software Version 2 (Biosoft). CI > 1, antagonism (A); CI = 1, additivity (Ad); CI < 1, synergy (S). Data are means ± SEM of three independent experiments, each carried out in triplicate. (c) Three-dimensional spheroids were treated for 24 h and IC_50_ values were determined using Origin Software, after the calculation of the effect of drugs on 3D spheroids volume by Matlab, as reported in Materials and Methods. Data are means ± SEM of five sample for each concentration (*n* = 2 independent experiments).

**Fig. 2:**
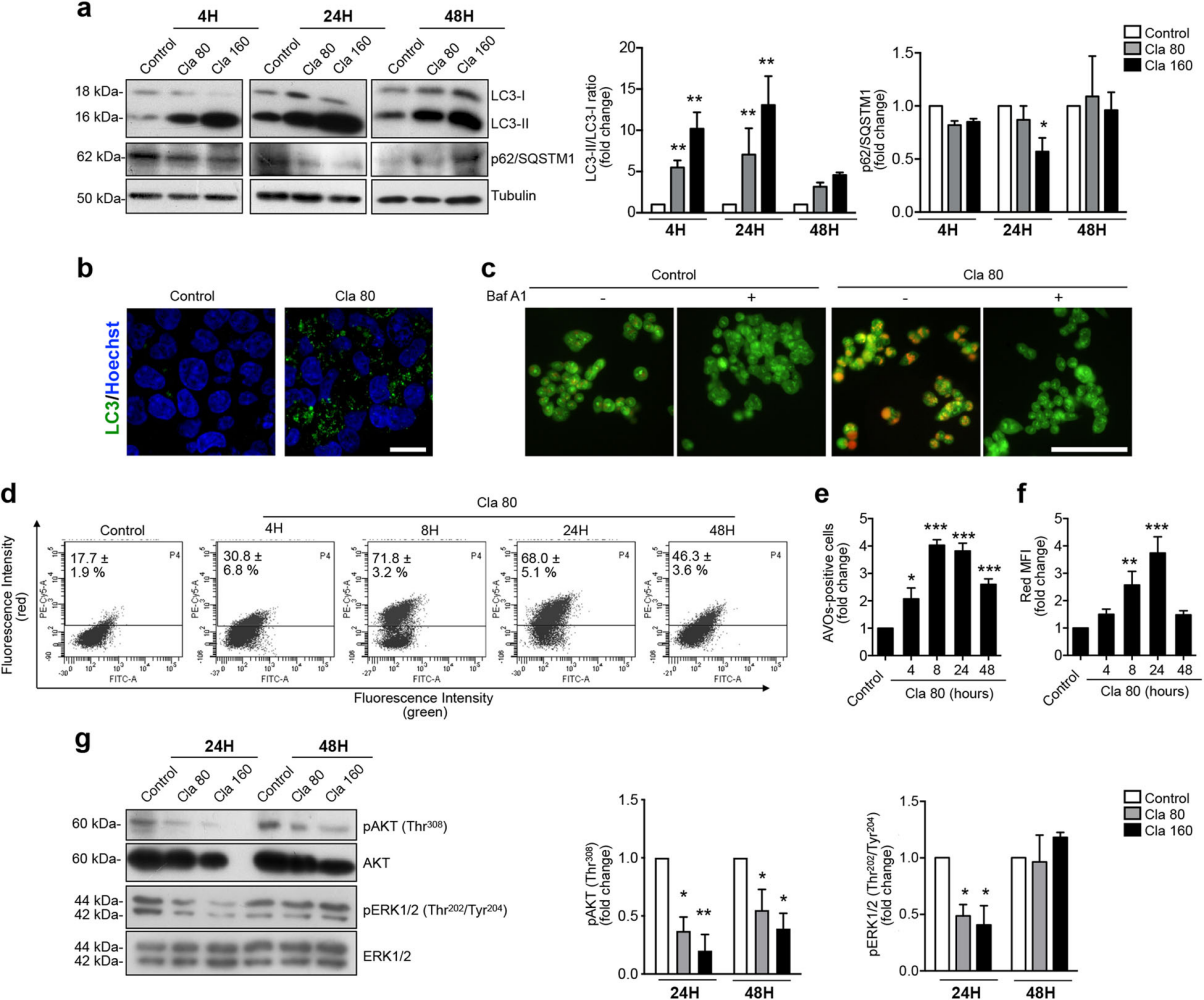
Clarithromycin modulates the autophagic flux in human CRC cell lines. **a** Time-course of LC3 and p62/SQSTM1 expression in HCT116 cells treated with Cla (80 and 160 µM) evaluated by western blot (WB). The corresponding densitometric results are given in the bar graphs (*n* = 4). **b** Expression of LC3 protein evaluated by immunofluorescence. HCT116 cells were treated for 24 h with Cla (80 µM). Representative fields from two independent experiments are shown (original magnification, ×400; scale bar, 20 µm). **c** Accumulation of acidic vesicular organelles (AVOs), which emitted bright red fluorescence, was investigated by acridine orange (AO) staining. HCT116 cells were treated with Cla (80 µM) or BafA1 (5 nM) or co-treated with Cla and BafA1, for 8 h. Representative fields from two independent experiments are shown (original magnification, ×200; scale bar, 100 µm). **d**–**f** Accumulation of AVOs was determined by AO staining and flow cytometry, after treating HCT116 cells with Cla (80 µM) for different time points (4–48 h). Quantification of AVOs is expressed as the percentage of AVOs-positive cells (indicated on the representative dot plots), and as fold change compared to control cells (**e**) and as the fold change of red mean fluorescence intensity (MFI) (**f**) in the bar graphs (*n* ≥ 4). **g** WB analysis of the protein levels of phospho-Akt^Thr308^ and phospho-ERK1/2^Thr202/Tyr204^ in HCT116 cells treated with Cla (80 and 160 µM) for 24 and 48 h. The membranes were probed with anti-Akt or anti-ERK1/2 antibodies. The corresponding densitometric results are given in the bar graphs (*n* = 3). Statistical significance for comparison of Cla-treated cells vs control cells was carried out with one-way ANOVA for (**a**, **e**, **f**, **g**; *P < 0.05; **P < 0.01, and ***P < 0.001.

The autophagic nature of the intracytoplasmic vacuoles obtained in HCT116 cells after Cla treatment was proven studying (i) the conversion of the soluble form of LC3, LC3-I, to the lipidated autophagosome-associated form, LC3-II and (ii) the amount of the autophagy cargo receptor p62/sequestome 1 (p62/SQSTM1), which is progressively degraded in the autolysosome during late autophagy^1,56^. Cla strongly increased the LC3-II/LC3-I ratio, in a dose- and time-dependent manner, with a maximum at 24 h of treatment (Fig. 2a). This effect was accompanied by a decrease of p62/SQSTM1 (Fig. 2a). At 24 h of Cla treatment, a characteristic punctate green fluorescent spot of LC3 was observed in the cytoplasm, a clear sign of induced autophagy, with presence of mature autolysosomes (Fig. 2b). Induction of autolysosome formation was confirmed by the accumulation of acidic vesicular organelles (AVOs), which was completely blocked by the autophagy inhibitor Bafilomycin A1 (BafA1) (Fig. 2c). However, at 48 h of Cla treatment, a decrease of LC3-II and a re-increase of p62/SQSTM1 were observed (Fig. 2a). This is suggestive of a late block of autophagic flux, which agrees with the reduction of the percentage of cells with vacuoles, of the number of vacuoles per cell, and with the increased size of vacuoles, observed at 48 h of treatment (Fig. 1d–g).

These findings led us to hypothesize that Cla initially facilitates autophagy, whereas at longer times the exhaustion of autophagic flux leads to autophagy block. To test this hypothesis, we quantified the time-course of Cla-induced AVOs formation by flow cytometry, measuring both the percentage of AVO-positive cells and the mean red fluorescence intensity (Red MFI), which is an indicator of AVOs’ acidity. Cla induced a rapid increase in the percentage of AVO-positive cells, the maximum levels being achieved after 8 h and maintained up to 24 h of treatment (Fig. 2d, e), which was accompanied by Red MFI increase (Fig. 2f). These data confirm the accumulation of autolysosomes in the cytosol of Cla-treated HCT116 cells, up to 24 h. However, the percentage of AVO-positive cells and, more evidently, red MFI decreased after 48 h (Fig. 2d–f), showing that AVOs’ acidification subsided after 48 h of Cla treatment, and autophagy declined.

We conclude that Cla initially induces autophagosome initiation and expansion, but subsequently causes a progressive engulfment of the process, with ensuing inhibition of autophagy.

Finally, we analyzed the signaling mechanisms implicated in the pro-autophagic effects of Cla, focusing on Akt and ERK1/2 phosphorylation^57,58^. Cla-reduced Akt phosphorylation both at 24 and 48 h. The decrease of ERK1/2 phosphorylation observed at 24 h was, on the contrary, relieved at 48 h (Fig. 2g).

### Clarithromycin modulates the PI3K/Akt pathway by targeting hERG1

**Fig. 3:**
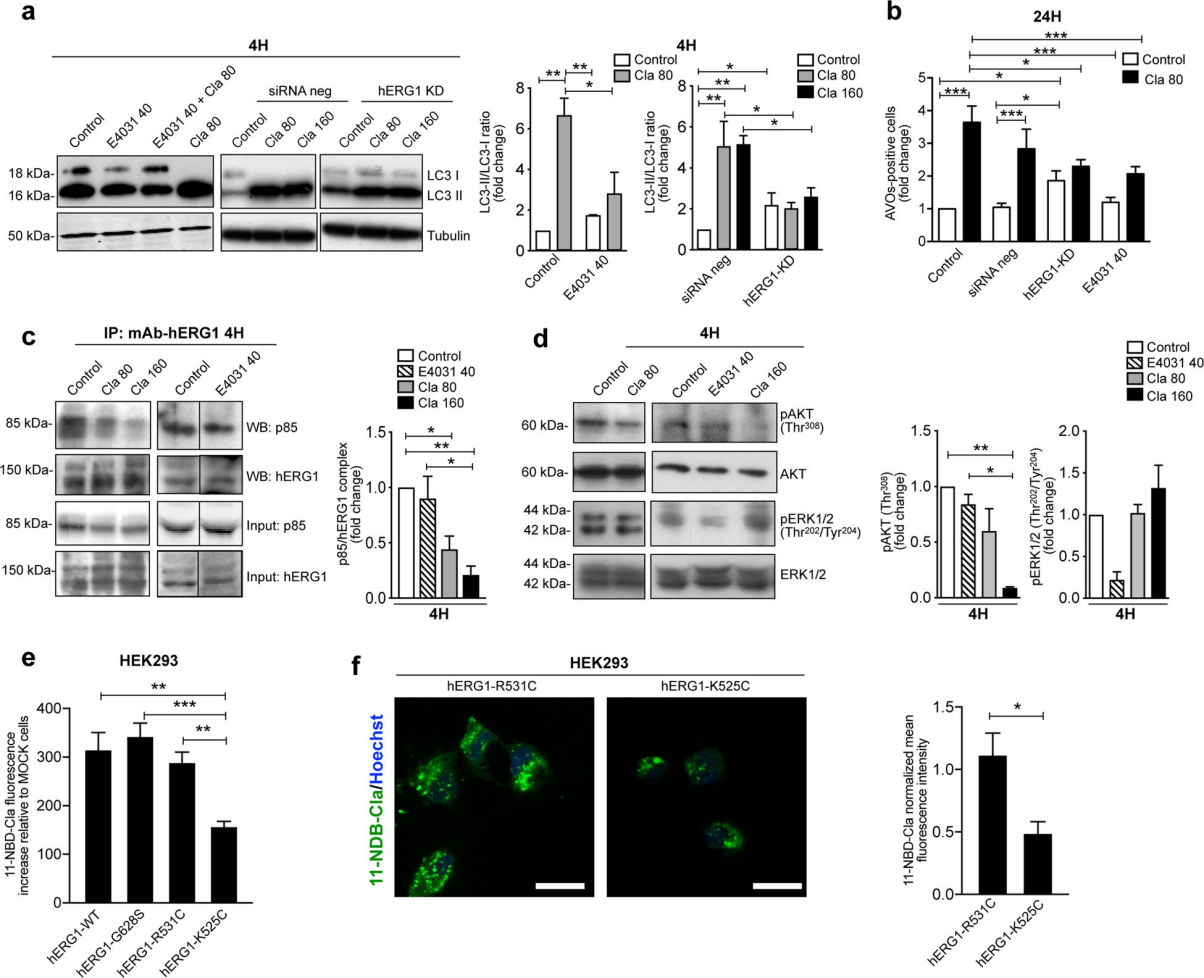
Clarithromycin modulates autophagy by targeting hERG1. **a** LC3 protein levels assessed by WB in HCT116 cells transfected for 24 h with specific α-*hERG1* siRNAs (hERG1-KD) or with siRNA negative control (siRNA neg), or of HCT116 wild type cells treated with E4031 (40 µM^59^), after 4 h Cla treatment (80 and 160 µM). The corresponding densitometric results are given in the bar graph (*n* = 3). **b** Accumulation of acidic vesicular organelles (AVOs) in the same cells as in (**a**), after 24 h of treatment with 80 μM Cla. Data are expressed as fold increase of AVOs-positive cells compared to HCT116 WT control-treated cells (*n* = 3). **c** Co-immunoprecipitation of hERG1 and the p85 subunit of PI3K from HCT116 cells treated with Cla (80 and 160 µM) or E4031 (40 µM) for 4 h. The corresponding densitometric results are given in the bar graph (*n* = 3). **d** Expression of phospho-ERK1/2^Thr202/Tyr204^ and phospho-Akt^Thr308^ in HCT116 cells treated as in (**c**) for 4 h. The membranes were re-probed with anti-ERK1/2 or anti-Akt antibodies, to normalize the amounts of phosphorylated corresponding proteins. The densitometric results are given in the bar graphs (*n* = 3). **e** Cla binding assay in HEK293 cells expressing hERG1 (hERG1-WT) and hERG1 mutants with different conformational state. The biding of fluorescently labeled 11-O-{3-[(7-nitro-2,1,3-benzoxadiazol-4-yl)amino]propyl}-6-O-methyl-erythromycin A (11-NBD-Cla) was determined, as detailed in Materials and Methods. The following hERG1 mutants were used: hERG-G628S, hERG1-R531C, and hERG1-K525C. Fluorescence intensities were normalized on total protein content, the intensity obtained in HEK293 MOCK-transfected cells was subtracted, and the obtained fluorescence intensity values were then normalized on the relative hERG1 expression in the different HEK293 transfected cells mutants according to data shown in ref. ^48^ (*n* = 3). **f** Confocal microscopy images obtained in the same cells and experimental conditions as in (**e**). Top focal plane of the z-stack images for HEK293 cells transfected with the hERG1-R531C and the hERG1-K525C mutants. Mean cell fluorescence measured at top section z-stack and normalized on the selected cell area are shown in the bar graph. The results are representative of three independent experiments (total number of cells analyzed for each group = 21) (scale bar, 20 µm). Statistical significance for the indicated comparisons was assessed with one-way ANOVA for (**a**–**e**), and with Student’s *t* test for (**f**); *P < 0.05, **P < 0.01, and ***P < 0.001.

A major Cla target in mammalian cell membranes is the K^+^ channel encoded by the (*hERG1*, also known as *Kv11.1*) ^35,36^, and hERG1 is often aberrantly expressed in cancers, including CRC^39–44^. Hence, the molecular basis of Cla’s effect was studied by manipulating hERG1 expression and function. First, we applied Cla after *hERG1* (normally expressed by HCT116 cells) was silenced with specific siRNAs^44^ (Fig. S3a) or blocking hERG1 current with the specific open channel blocker E4031 at 40 μM^59^. *hERG1* silencing significantly increased the basal accumulation of LC3-II (Fig. 3a) and the basal number of AVO-positive cells (Fig. 3b), suggesting the involvement of hERG1 protein in the control of CRC cell autophagy. Moreover, both *hERG1* silencing and E4031 reduced the effects of Cla on LC3 conversion (Fig. 3a), AVOs (Figs. 3b and S3b) and vacuoles (Fig. S3c) formation. These data indicate that Cla exerts its effect on CRC cell autophagy, by modulating hERG1.

We previously showed that hERG1 channels stimulate the PI3K/Akt pathway by forming a macromolecular complex with the p85 subunit of PI3K, in human CRC cells^44^. To test whether this mechanism was implicated in the Cla-dependent decrease of Akt phosphorylation, we carried out co-IP experiments in which hERG1 was immunoprecipitated and the blot was revealed with anti-p85 PI3K antibodies. After 4 h of treatment, Cla inhibited the formation of the complex between hERG1 and p85 (Fig. 3c). This effect was paralleled by the reduction of phosphorylated Akt, with no effect on ERK 1/2 phosphorylation (Fig. 3d). On the contrary, E4031 did not affect Akt phosphorylation, while decreased the amount of phosphorylated ERK1/2 (Fig. 3d). Noteworthy, treating HCT116 cells with E4031 did not affect the formation of the hERG1/PI3K complex (Fig. 3c). These data indicate that Cla affects the PI3K/Akt pathway by impairing the functional interaction between hERG1 and the p85 subunit of PI3K, and that these effects are different from those exerted by hERG1 blockers.

### Clarithromycin affects the balance of hERG1 conformational states to modulate autophagy

To further investigate the molecular basis of the above signaling effects, we first performed both an in vitro drug-cell binding assay and confocal imaging using fluorescently labeled Cla (11-NBD-Cla^52^). We used HEK293 cells transfected with wild-type hERG1 (hERG1-WT), as well as hERG1 mutants with different steady-state activation properties^48,60^. MOCK-transfected HEK293 cells were considered as negative control. In particular, we used (i) the non-conducting hERG1-G628S, whose gating properties are similar to the hERG1-WT’s, (ii) hERG1-K525C, in which the voltage-dependent transition to the open state is favored, and (iii) hERG1-R531C, in which the voltage-dependent transition to the open state is impaired. More specifically, at the typical resting membrane potential (*V*_rest_) of HEK293 cells transfected with the different constructs, approximately 30% of hERG1-K525C channels reside in the active state (*V*_rest_ ~−60 mV), whereas hERG1-WT, hERG1-G628S, and hERG1-R531C (with *V*_rest_ ranging between −40 and −50 mV) essentially reside in the closed state^48^. Whereas no significant differences in Cla binding were detected between cells transfected with hERG1-WT, or hERG1-G628S, or hERG1-R531C, transfection with hERG1-K525C led to a reduced affinity to Cla (Fig. 3e, f and Fig. S4). These results suggest that the propensity of hERG1-K525C to reside in the open state hinders Cla binding, in agreement with previous pharmacological results indicating that Cla tends to bind to closed hERG1 channels^35^.

**Fig. 4:**
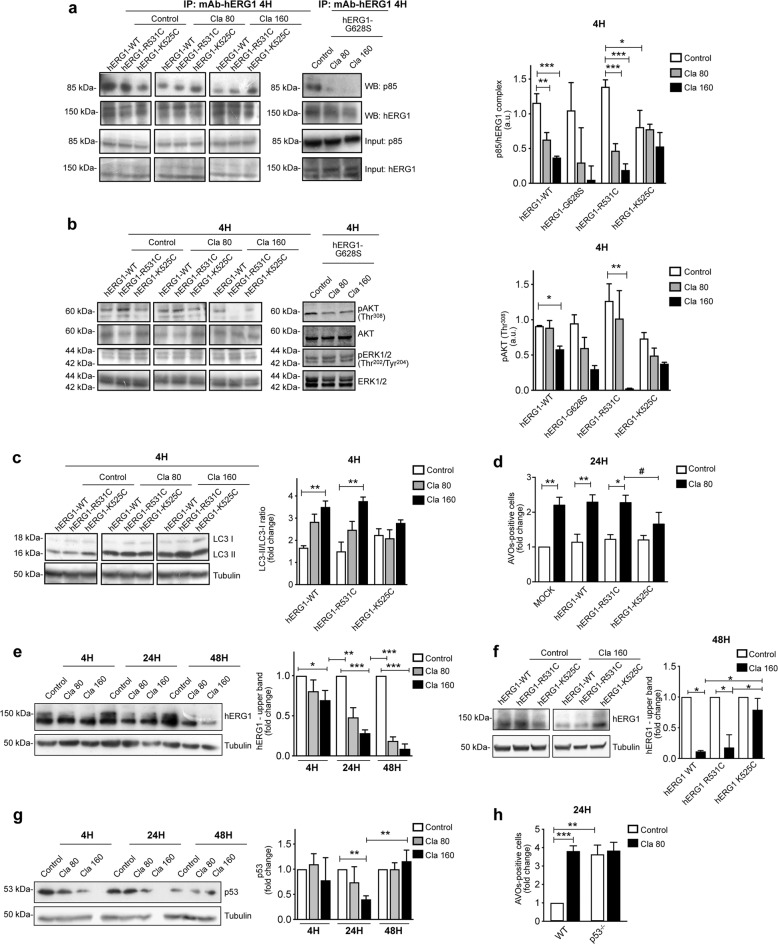
Clarithromycin modulates autophagy by inhibiting the hERG1/PI3K complex formation and the downstream Akt pathway: role of hERG1 conformational states. **a** Co-immunoprecipitation of hERG1 and the p85 subunit of PI3K in HCT116 cells expressing hERG1 (hERG1-WT), or hERG1-K525C, or hERG1-R531C, or hERG1-G628S and treated with Cla (80 and 160 µM) for 4 h. The corresponding densitometric results are given in the bar graph (*n* = 3). **b** Expression of phospho-ERK1/2^Thr202/Tyr204^ and phospho-Akt^Thr308^ in HCT116 cells expressing different hERG1 mutants treated as in (**a**). The membranes were re-probed with anti-ERK1/2 or anti-Akt antibodies, to normalize the amounts of phosphorylated corresponding proteins. The densitometric results are given in the bar graphs (*n* = 3). **c**, **d** LC3 expression (**c**) and accumulation of AVOs (**d**) in HCT116 cells expressing hERG1 (hERG1-WT), or hERG1-K525C, or hERG1-R531C after 4 and 24 h of treatment with Cla (80 µM), respectively. **c** LC3 levels were assessed by WB and the corresponding densitometric results are given in the bar graph (*n* = 3). **d** Quantification of AVOs is expressed as fold increase of AVOs-positive cells compared to HCT116 MOCK cells (*n* = 3). **e** WB analysis of hERG1 protein expression in HCT116 cells were treated with Cla (80 and 160 µM) for 4, 24, and 48 h. **f** WB analysis of hERG1 protein expression in HCT116 cells transfected with hERG1-WT, or hERG1-K525C, or hERG1-R531C and treated for 48 h with Cla (160 µM). The corresponding densitometric analysis is given in the bar graphs (*n* = 3). **g** WB analysis of p53 protein expression in HCT116 cells treated with Cla as in (**e**). The corresponding densitometric analysis is given in the bar graph (*n* = 3). **h** Accumulation of AVOs in HCT116 p53^−/−^ cells after 24 h of treatment with Cla (80 µM). Quantification of AVOs is expressed as in (**d**) (*n* = 3). Statistical significance for the indicated comparisons was assessed with one-way ANOVA for (**a**–**h**); *P < 0.05, **P < 0.01, and ***P < 0.001; in panel **d** the comparison between the data obtained with Cla in hERG1R31C- and hERG1-K525C- transfected cells was assessed by Student’s *t* test, ^#^*P* < 0.05.

Next, we tested the biological effect of Cla in HCT116 cells endogenously expressing hERG1, and transfected with either hERG1-WT or the above hERG1 mutants. The plasma membrane abundance of the different hERG1 constructs was determined by flow cytometry (Fig. S5). Transfecting the cells with hERG1-WT or hERG1-G628S did not modify the basal effect produced by Cla on HCT116. On the contrary, transfecting the cells with hERG1-K525C impaired the inhibition exerted by Cla both on hERG1/p85 complex formation, and Akt phosphorylation (Fig. 4a, b), whereas these effects were increased in cells transfected with hERG1-R531C (Fig. 4a, b). Consistently, both LC3-II accumulation at 4 h and AVOs accumulation at 24 h were scarcely affected when Cla was added to HCT116 cells expressing hERG1-K525C, compared to cells transfected with either hERG1-WT or hERG1-R531C (Fig. 4c, d; the data relative to the G628S mutant are shown in supplementary Fig. S6). The simplest interpretation of our results is that hERG1-K525C, in which the transition to the open state is energetically favored, and thus the ratio between open and closed channels is larger, hampers Cla binding and thus impairs the drug’s effects on autophagy. Our interpretation is consistent with the observation that the biological effects of Cla on cells expressing hERG1-K525C are analogous to those produced by E4031, which blocks hERG1 currents by obstructing the open channel.

Finally, long term (48 h) exposure to Cla produced a significant reduction of the amount of the hERG1 protein (Fig. 4e). This decrease could not be traced back to an autophagy-mediated protein degradation, since the expression of another K+ channel (K_Ca_ 3.1) expressed at high levels in HCT116 cells was not affected by Cla treatment (Fig. S7). We attribute the long term decrease in hERG1 expression to a specific effect of Cla, which could favor hERG1 degradation, by sequestering the channel in the closed state. In agreement with this hypothesis, Cla did not produce a decrease of hERG1 in cells transfected with hERG1-K525C, compared to cells transfected with either hERG1-WT or hERG1-R531C (Fig. 4f).

Considering its relevant role in autophagy^61,62^, we tested whether p53 could be involved in the effects of Cla. After 4 and 24 h Cla reduced p53, which went back to its basal amount after 48 h of treatment (Fig. 4g). Notably, Cla had no effect on the percentage of AVOs-positive cells in HCT116 cells in which p53 was deleted (HCT116 p53^−/−^) (Fig. 4h and S8). Overall, these findings suggest that p53 is related to the modulation of autophagy induce by Cla.

### Prolonged treatment with Clarithromycin alters cell proliferation and triggers apoptotic cell death in CRC cells

**Fig. 5:**
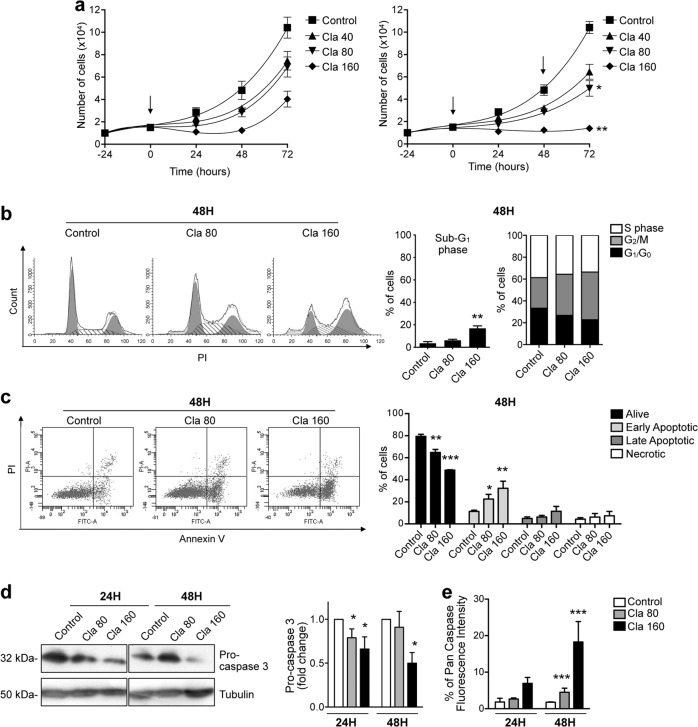
Effects of Clarithromycin on proliferation, cell cycle distribution, and apoptosis of HCT116 cells. **a** Effects of Cla (40, 80, and 160 µM) on proliferation (expressed as the number of alive, trypan blue-negative, cells) of HCT116 cells, after a single (arrow; left panel) and a double treatment (arrows; right panel) (*n* = 3, each carried out in triplicate). The reported significance levels (asterisks) refer to the difference between Cla double treatment and Cla single treatment. Other statistical analyses are shown in Table S3. **b**, **c** HCT116 cells were treated with Cla (80 and 160 µM). After 48 h of treatment, cells were harvested and stained for flow cytometric analysis of cell cycle distribution (**b**) and apoptosis (**c**). Representative histograms of cell cycle analysis and relative percentages of gated cells at sub-G_1_, G_1_/G_0_, S, and G_2_/M phases are reported in (**b**). Representative dot plots of Annexin-V/PI analysis and relative percentages of gated cells for alive (annexin V-negative and PI-negative), necrotic (annexin V-negative and PI-positive), early apoptotic (annexin V-positive and PI-negative), and late apoptotic (annexin V-positive and PI-positive) HCT116, are reported in (**c**) (*n* = 4). Data relative to 24 h are in Fig. S8. **d** WB analysis of the protein levels of pro-caspase 3 in HCT116 cells treated with Cla (80 and 160 µM) for 24 and 48 h. The membranes were re-probed with an antitubulin antibody to normalize pro-caspase data. The corresponding densitometric results are given in the bar graphs (*n* = 3). **e** Activation of caspases following Cla treatment determined by the Generic Caspase Activity Assay Kit and flow cytometric analysis. Quantitative analysis of Pan Caspase Fluorescence Intensity in HCT116 cells, treated for 24 and 48 h with Cla (80 and 160 µM) is reported (*n* = 3). Statistical significance was assessed with one-way ANOVA for (**a**–**d**); *P < 0.05; **P < 0.01, and ***P < 0.001.

Because of the undisputed role of p53 in the control of cell growth vs apoptosis^63^, we moved to analyze the effects of Cla on cell proliferation and apoptosis of human CRC cells. After 24-h, Cla reduced the viability of CRC cells in a dose-dependent manner. An example, relative to HCT116 cells, is shown in Fig. S1. The IC_50_ values observed on the different CRC cell lines are given in Table 1a. Values ranged from 80.0 to 154.4 μM. Long-term exposure to a single treatment with 40, 80, or 160 μM Cla (i.e., the IC_25_, IC_50_, and IC_75_ concentrations, respectively) reduced HCT116 cell proliferation, although did not completely abolished it (Fig. 5a). Inhibition of cell proliferation was potentiated when Cla was re-added to the cells. In particular, 160 μM Cla, re-added after 48 h of incubation, produced an arrest of cell proliferation at 72 h (Fig. 5a). Similar effects were obtained in LS174T cells (Fig. S9).

Cla also led to an increase in the percentage of G_2_/M cells after 24 h (Fig. S10a) and, even more, 48 h of treatment (Fig. 5b). A 48-h treatment with the highest dose of Cla increased the percentage of cells in sub-G_1_ phase (Fig. 5b), further suggesting that Cla might trigger apoptosis. Indeed, after 48 h, a high percentage of cells in early apoptosis (annexin-V-positive/PI-negative) was detected (Figs. 5c and S10b). Consistently, Cla produced a reduction of pro-caspase 3 levels (Fig. 5d), and a concomitant activation of caspases (Fig. 5e), more evident after 48 h.

### Clarithromycin induces apoptotic cell death via regulation of hERG1 and p53

**Fig. 6:**
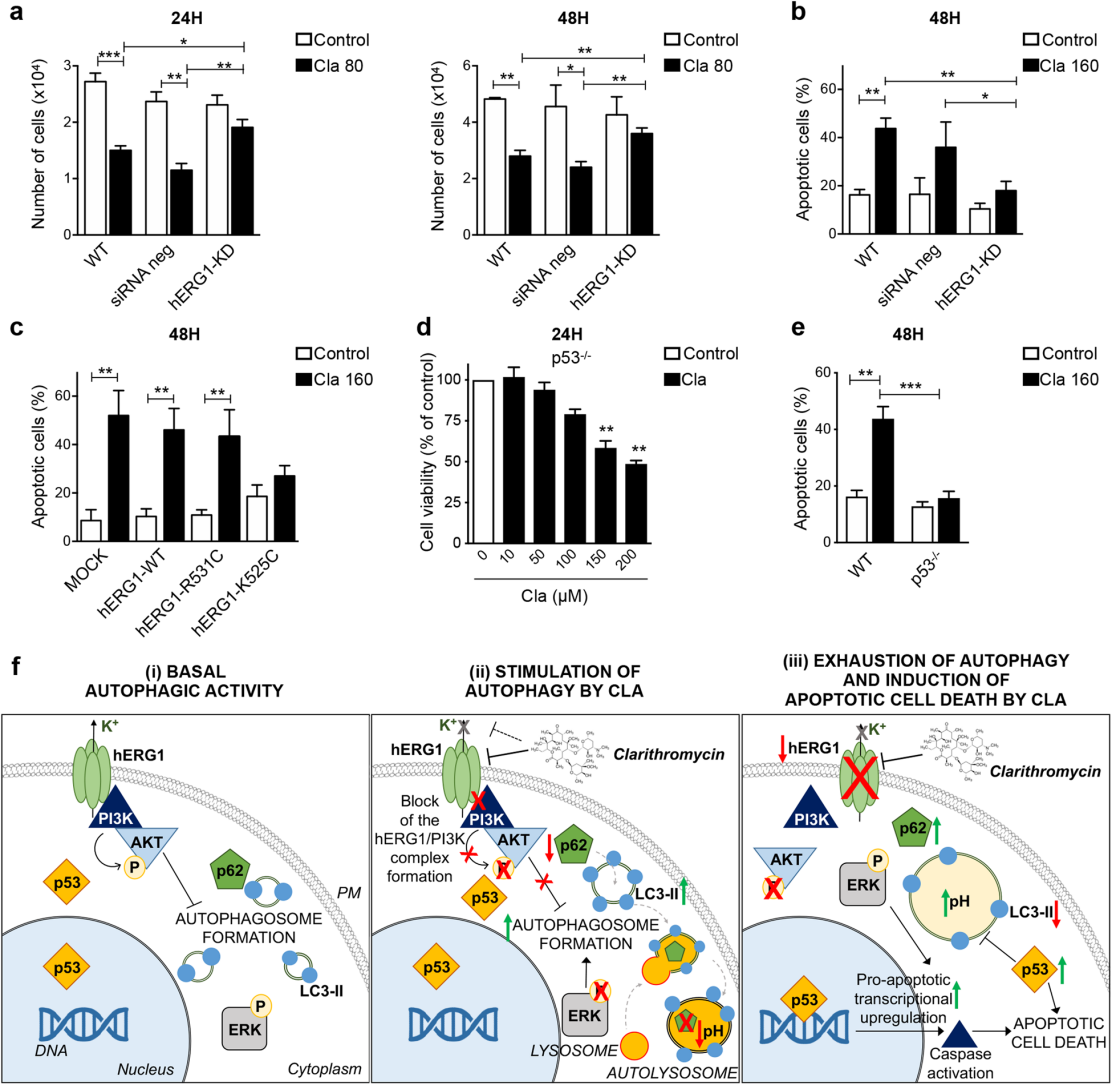
The pro-apoptotic effect of Clarithromycin is mediated by hERG1 and p53. **a**, **b** HCT116 cells were silenced for *hERG1* as in Fig. 3a. Twenty-four hours after siRNA transfection, cells were treated with Cla (80 and 160 µM). After 24 and 48 h of incubation, cells were harvested and cell viability (**a**) and apoptosis (**b**) were assessed. Cell viability is expressed as the number of alive, trypan blue-negative, cells. Percentage of apoptotic (early apoptotic plus late apoptotic) cells was determined by the Annexin-V/PI staining (*n* = 3). **c** Percentage of apoptotic (early apoptotic plus late apoptotic, determined by the Annexin-V/PI staining) HCT116 cells expressing either hERG1-WT, or hERG1-K525C, or hERG1-R531C, after 48 h of treatment with Cla (160 µM) (*n* = 3). **d** Cell viability of HCT116 p53^−/−^ cells after 24 h of treatment with Cla (0–200 µM). Data are reported as percentage of control cells (*n* = 4, each carried out in triplicate). **e** Percentage of apoptotic (early apoptotic plus late apoptotic, determined by the Annexin-V/PI staining) HCT116 p53^−/−^ cells treated with Cla (160 µM) for 48 h (*n* = 3). Statistical significance was assessed with a one-way ANOVA test for (**a**–**f**); *P < 0.05; **P < 0.01, and ***P < 0.001. **f** Schematic diagram summarizing the hypothesized mode of action of Cla in human CRC cells. (**i**) Basal autophagic activity: hERG1 is highly expressed on the plasma membrane (PM) of HCT116 cells and forms a macromolecular complex with the p85 regulatory subunit of PI3K. The hERG1/PI3K complex leads to Akt phosphorylation, which regulates autophagy. (**ii**) Stimulation of autophagy by Cla: Cla inhibits the hERG1/PI3K complex formation, which leads to reduced Akt and ERK1/2 phosphorylation, and increases autophagic activity. This effect is correlated to an increase in LC3-II conversion and accumulation, a reduction of p62/SQSTM1 and the accumulation of autolysosomes. (**iii**) Exhaustion of autophagy and induction of apoptotic cell death by Cla: prolonged treatment with Cla leads to hERG1 degradation, and an impairment of the autophagic process, characterized by increased vacuole size and intra-vacuolar alkalinization. As a consequence, expression of p62/SQSTM1 and phosphoERK1/2 recovers and LC3-II accumulation decreases. These effects are also correlated to a late recovery of p53, responsible for caspases activation and apoptotic cell death.

Next, we tested whether the pro-apoptotic effect of Cla was also related to hERG1 modulation. As shown in Table 1a, Cla scarcely affected HEK293 cells (IC_50_ > 200 μM; Fig. S11), whereas it exerted a strong cytotoxic effect on HEK293 cells transfected with the *hERG1* cDNA (HEK-hERG1 cells; IC_50_ was 92.4 ± 4.9 μM). The knockdown of *hERG1* in HCT116 cells by siRNA, resulted in a significant decrease in Cla-mediated induction of cytotoxicity (Fig. 6a) and apoptotic cell death in HCT116 cells (Figs. 6b and S12a). Furthermore, the pro-apoptotic effect of Cla was evident in MOCK-transfected cells, as well as in cells transfected with hERG1-WT and hERG1-R531C, whereas it was negligible in cells transfected with hERG1-K525C, suggesting that the conformational state of the channel is also implicated in the pro-apoptotic effects of Cla (Fig. 6c). Finally, Cla scarcely affected cell viability (Fig. 6d and Table 1a) and produced a lesser percentage of apoptotic cells (Figs. 6e and S12b) in HCT116 p53^−/−^ cells, in agreement with the involvement of p53 in the pro-apoptotic effects of Cla.

A schematic diagram of our interpretation of the results presented so far is given in Fig. 6f.

### Clarithromycin potentiates the cytotoxic effects of 5-Fluorouracil in vitro and in vivo

We next tested whether Cla affected the antineoplastic effects of chemotherapeutic drugs commonly used for CRC treatment: irinotecan (CPT-11), 5-Fluorouracil (5-FU), cisplatin (Cis), and oxaliplatin (Oxa)^64^. CPT-11 and 5-FU exerted considerable cytotoxic effects on HCT116 cells at low doses (Tables 1b and S13a), whereas Cis and Oxa affected HCT116 cells’ viability only when used at high concentrations, as previously demonstrated^50^. Furthermore, Cla was synergized with both CPT-11 and 5-FU; and this effect was more prominent when the drugs were combined at their IC_25_ values (Tables 1b and S13b). On the contrary, Cla was antagonistic with Cis and Oxa when the drugs were combined at their IC_50_ values (Table 1b).

**Fig. 7:**
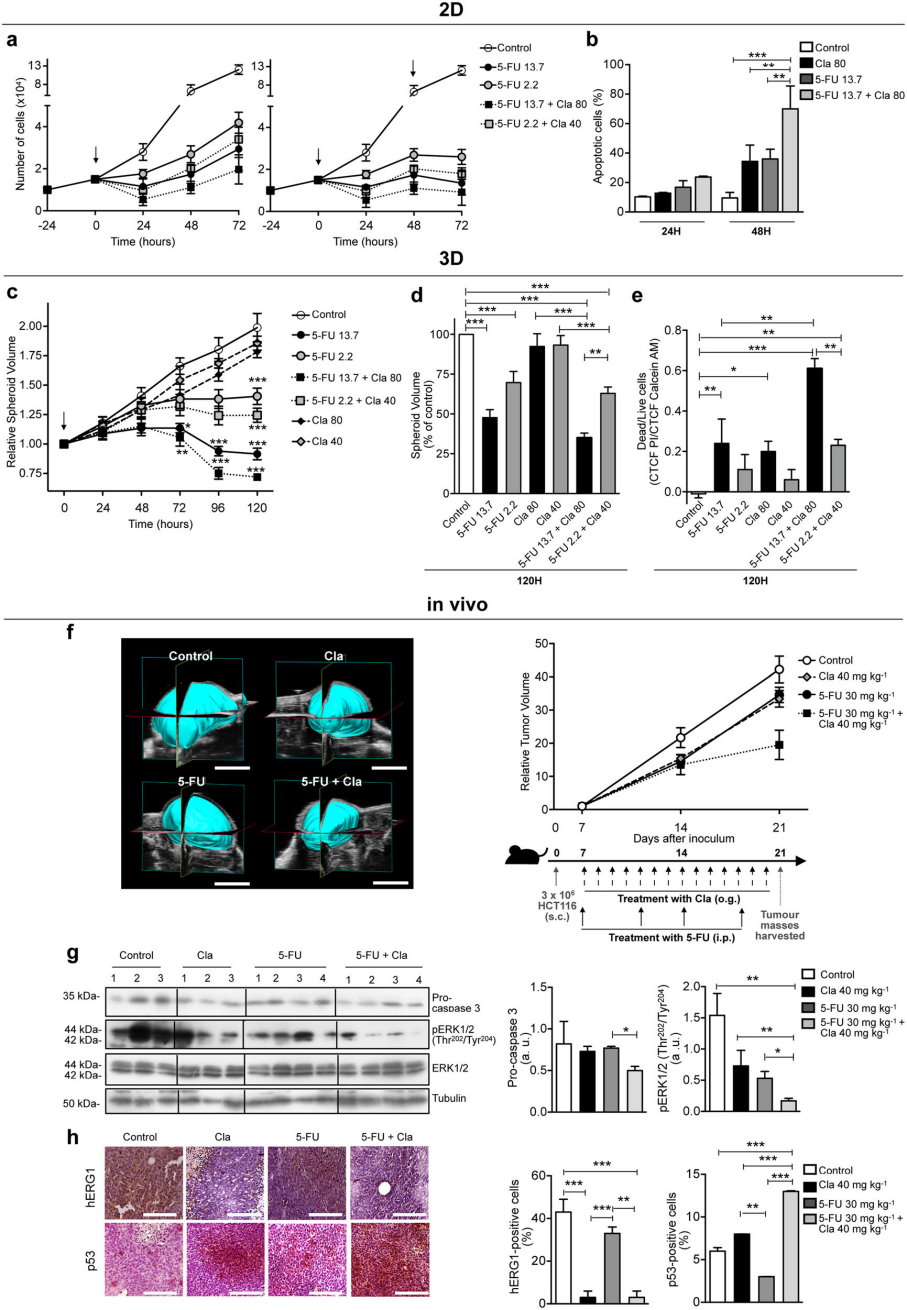
Clarithromycin potentiates the cytotoxic effect of 5-Fluorouracil (5-FU) in 2D, 3D cell cultures, and in vivo CRC models. **a** Effects of 5-FU on proliferation of HCT116 cells, alone (IC_25_: 2.2 µM; IC_50_: 13.7 µM) or in combination with Cla (IC_25_: 40 µM; IC_50_: 80 µM). Experiments were carried out as described for Fig. 4a. Data points give the number of trypan blue-negative cells (*n* = 3, each carried out in triplicate). Full statistics are reported in Table S3. **b** Percentages of apoptotic (early apoptotic plus late apoptotic from Annexin V/PI staining) HCT116 cells treated as in (**a**) for 24 and 48 h (*n* = 3). **c** Time course of spheroids’ volume relative to their initial volume (time 0) for the indicated conditions. Drugs were added at their IC_50_ and IC_25_ values, obtained in 2D cultures (*n* = 3; 8 samples for each condition). Spheroids’ photographs, taken with an inverted microscope (Nikon Eclipse TE300, with a ×10 objective), were used to calculate spheroid volumes as in ref. ^54^ by SpheroidSizer1_0, a MATLAB-based and open-source software (MATLAB 2015a, MathWorks Inc.). The reported significance values refer to the comparison between each condition and control cells. **d**, **e** Volumes (**d**) and cell viability (**e**) of spheroids treated as in (**c**) for 120 h. Spheroid volumes are reported as the percentage of control (*n* = 3; 8 samples for each condition). Cell viability was assessed by the live/dead imaging (see Materials and Methods) (*n* = 2; 6 samples for each condition). **f** Representative 3D reconstruction of tumor masses, performed in B-Mode imaging with VevoLAZR-X (scale bar, 5 mm) (left panel). Time course of tumor growth relative to the volume determined at the beginning of treatment (day 7 after the inoculum) (right panel). One week after subcutaneous (s.c) inoculation (3 × 10^6^ HCT116 cells/flank), mice were treated for two weeks with saline (control group), Cla (40 mg kg^−1^, twice daily by oral gavage (o.g.), 5-FU (30 mg kg^−1^, twice a week intraperitoneally (i.p.)), or the combination of Cla plus 5-FU (*n* = 2 mice; *n* = 4 tumor masses, for each for each experimental group), as reported in the schematic representation of treatment regime. Statistical analysis was performed with one-way ANOVA: Cla + 5-FU vs. control mice, *P* = 0.019; Cla + 5-FU vs. Cla mice, *P* = 0.033; Cla + 5-FU vs. 5-FU mice, *P* = 0.046. **g** Expression of phospho-ERK1/2^Thr202/Tyr204^ and pro-caspase 3 in tumor masses obtained from mice treated as in (**f**). Membranes were re-probed with anti-ERK1/2 or antitubulin antibodies, to normalize data. The corresponding densitometric analyses are given in the bar graphs (*n* = 4 for each experimental group). **h** IHC analysis of hERG1 and p53 in tumor masses of HCT116 tumor xenografts of mice treated as in (**f**). Representative images are reported (original magnification, ×400; scale bar, 100 µm). The percentages of cells positive for either hERG1 or p53 are reported in the bar graph (*n* = 2 mice, *n* = 4 tumor masses for each experimental group). Statistical significance was assessed with one-way ANOVA for (**a**–**h**); *P < 0.05; **P < 0.01, and ***P < 0.001.

Considering that the strongest synergy was obtained combining Cla and 5-FU, we further investigated the effects of this drug combination. A long-term treatment with Cla and 5-FU led to inhibition of HCT116 cell proliferation and to induction of apoptosis (Figs. 7a, b and S14). Both effects were stronger than those produced by the single drugs (Fig. 7a).

We also quantified the effects of 5-FU and Cla (tested at the IC_50_ and IC_25_ values determined in 2D cultures) on the growth of HCT116 cells cultured in 3-dimensions (3D) as spheroids. Both the volumes of the spheroids and the dead to live ratio (determined by Calcein/PI staining) were determined. 3D cultures are commonly less sensitive to chemotherapeutic drugs^54^ compared to 2D cultures. Both 5-FU and Cla (Table 1c and Fig. S15) were less effective on 3D compared to 2D cultures, as expected^54^. Nevertheless, a substantial reduction in the volumes of spheroids accompanied by the highest number of dead cells was observed after 120 h of combined treatment with 5-FU and Cla (Figs. 7c–e and S16).

The effects of Cla on tumor growth, Akt and ERK1/2 phosphorylation, caspase activation and autophagic markers (LC3 and p62/SQSTM1) in vivo, in a CRC xenograft model, have been reported elsewhere^55^. Here, we studied the synergistic effect of Cla with 5-FU in the same CRC xenograft model. In this, the combination of Cla (administered twice a day for 14 days, at a dose of 40 mg kg^−1^) with subclinical doses of 5-FU (30 mg kg^−1^, twice a week for 14 days^20^) strongly potentiated the inhibitory effect of 5-FU on tumor growth (Fig. 7f). This effect was accompanied by a reduction of procaspase 3 levels and ERK1/2 phosphorylation (Fig. 7g), which is known to be related to the action of 5-FU^65^, as well as a reduction of hERG1 expression and accumulation of p53 (Fig. 7h).

## Discussion

We provide evidence in CRC that Cla exerts antiproliferative activity and enhances the antitumor effects of chemotherapeutic drugs, through a complex modulation of autophagy brought about by an hERG1–dependent regulation of the PI3K/Akt pathway and p53.

In CRC, we previously found that hERG1, once activated by integrin-mediated adhesion, forms a macromolecular complex with the p85 regulatory subunit of PI3K, which leads to Akt phosphorylation^44^. In fact, Cla turned out to inhibit formation of such macromolecular complex, causing a reduction of both Akt and ERK1/2 phosphorylation. As a consequence, the autophagic steady-state was shifted toward an increased autophagic activity. The latter was witnessed by an increase in the percentage of vacuolated cells, the conversion of LC3-I to LC3-II, the reduction of the autophagy cargo p62/SQSTM1, the accumulation of AVOs and the acidification of autolysosomes. However, after longer incubation times, we observed an increased vacuole size, and decreased autolysosome acidity, suggestive of engulfment of the autophagic process. As a consequence, the autolysosome-mediated degradation was blocked, both p62/SQSTM1 expression and ERK1/2 phosphorylation recovered, and hence the autophagic flux was inhibited. These effects were followed by apoptotic cell death, presumably sustained by p53 and caspases activation (Fig. 6f).

That hERG1 is implicated in the cellular mechanisms triggered by Cla is proven by the fact that *hERG1* knockdown impairs Cla-induced autophagy, and also suggested by the observation that channel degradation took place after 48 h of treatment with the macrolide. The progressive decrease of hERG1 amount probably contributes to autophagy engulfment. Nonetheless, *hERG1* knockdown per se induces autophagy, as was evidenced by an increase of LC3-II as well as of AVOs-positive cells. These results suggest a complex role of hERG1 in basal autophagy, which warrants future investigations.

We sought to better define how hERG1 regulated the Cla-dependent autophagy, by using different hERG1 constructs and blockers. The simplest interpretation of our results is that hERG1 interaction with p85, and the ensuing Akt phosphorylation, are favored when the channel is in the closed conformation, and that such interaction is antagonized by Cla binding. This conclusion is supported by the following observations: (i) the binding of Cla is independent on hERG1 conduction (see the effects on the G628S mutant) and is favored when hERG1 is in a closed conformation (see the effects on the R531C mutant) (Fig. 3e, f); (ii) E4031 alone, which targets the open channel^66^ has different effects compared to Cla and to *hERG1* knockdown, since it does not affect either the hERG1/PI3K complex (Fig. 3c) or Akt phosphorylation (Fig. 3d). On the contrary, E4031 decreases ERK1/2 phosphorylation (Fig. 3d), an effect we attribute to hERG1 ion conduction, as previously discussed in^43–48^. (iii) Cla is less effective in cells expressing hERG1-K525C and in cells treated with E4031; both these conditions tend to increase the steady-state fraction of open channels, although for different reasons; (iv) the effect of Cla is facilitated by expression of channels that have a low steady-state probability of opening at the normal V_rest_ of CRC cells, such as hERG1-WT and hERG1-R531C.

Numerous studies on different cancer preclinical models^23–31^ have addressed the role of Cla as an autophagy inhibitor, without clarifying the underlying molecular mechanism. We here demonstrate that the Cla-dependent modulation of autophagy in human CRC cells is biphasic: Cla initially stimulates autophagosome initiation and expansion, which is related to a decrease in both Akt and ERK1/2 phosphorylation. A decreased ERK activity has been shown to induce autophagic flux in other types of cancer cells^67,68^. Subsequently, Cla causes a progressive autophagy exhaustion and hence inhibition of the autophagic flux, as witnessed by the re-increase of pERK levels. The simultaneous induction and blockade of autophagy by a single agent was recently reported for other compounds, such as the tacrine–melatonin heterodimer C10^69^. The late impairment of autophagosome-mediated degradation is cytotoxic for CRC cells, as it triggers apoptotic cell death (Fig. 6f), as shown by the late increase of p53 levels. Although we did not deepen the complex role of p53 in autophagy^70^, our data obtained in p53^−/−^ cells suggest a supporting role of p53 in the pro-autophagic effects of Cla (Figs. 4h and S7).

From a translational standpoint, the anti-cancer activity of Cla was higher when the macrolide was administered in combination with CPT-11 and 5-FU, two of the most used and effective chemotherapeutic drugs for CRC treatment, which however, can lead to the development of drug resistance^71^, which imposes interruption of the therapeutic regimen^72^. Importantly, the synergic effect of Cla and 5-FU was particularly powerful in vivo, where we found a remarkable reduction of tumor size. This effect was accompanied by a strong decrease of hERG1 expression, of ERK1/2 phosphorylation (consistent with the effects of 5-FU on this signaling pathway^65^, and the role of ERK1/2 in modulating chemoresistance to 5-FU^73^) and increased caspases activity and p53 expression, which indicates that the in vivo effects of Cla mirror those observed in vitro. Interestingly, Cla did not synergize, but was antagonistic with Cis and Oxa which we attribute to the completely different mechanism that underlies the effect of platinum-based drugs^50^.

hERG1 is highly expressed in aggressive primary CRCs (Figs. S17 and S18)^74^, Cla, which is already used in the clinical setting^75^, could be proposed for CRC therapy. Furthermore, its effects on hERG1 may open the way to the development of further drugs targeting different hERG1conformational states to regulate autophagy in cancer.

## Supplementary information

Supplementary figure and table legends

Supplementary Figure S1

Supplementary Figure S2

Supplementary Figure S3

Supplementary Figure S4

Supplementary Figure S5

Supplementary Figure S6

Supplementary Figure S7

Supplementary Figure S8

Supplementary Figure S9

Supplementary Figure S10

Supplementary Figure S11

Supplementary Figure S12

Supplementary Figure S13

Supplementary Figure S14

Supplementary Figure S15

Supplementary Figure S16

Supplementary Figure S17

Supplementary Figure S18

Supplementary Table S1

Supplementary Table S2

Supplementary Table S3

## Data Availability

All the data needed to evaluate the conclusions in the paper are present in the paper or in the Supplementary Materials.
