# Correction: Clarithromycin inhibits autophagy in colorectal cancer by regulating the hERG1 potassium channel interaction with PI3K

**DOI:** 10.1038/s41419-020-2407-2

**Published:** 2020-03-30

**Authors:** Giulia Petroni, Giacomo Bagni, Jessica Iorio, Claudia Duranti, Tiziano Lottini, Matteo Stefanini, Goran Kragol, Andrea Becchetti, Annarosa Arcangeli

**Affiliations:** 1 Department of Experimental and Clinical Medicine, University of Firenze, Viale GB Morgagni 50, 50134 Firenze, Italy; 2 Fidelta Ltd., Prilaz baruna Filipovića 29, 10000 Zagreb, Croatia; 3 Department of Biotechnology and Biosciences, University of Milano-Bicocca, Piazza della Scienza 2, 20126 Milano, Italy

**Keywords:** Gastrointestinal cancer, Autophagy, Cell death

Correction to: *Cell Death and Disease*

10.1038/s41419-020-2349-8 published online 2 March 2020

The financial support for this Article was not fully acknowledged. The acknowledgements should have included the following:

We thank M. Lulli (University of Florence, Italy) for acquiring images of immunofluorescence-labeled cells. This work was supported by grants from Associazione Italiana per la Ricerca sul Cancro (#15627, #21510 and #19766 to A.A.); PAR FAS—Linea di Azione 1.1—Azione 1.1.2—Bando FAS Salute. 2014 (DD 4042/ 2014) Project OMITERC to A.A.; FAR 2018 to A.B.

