# Supplementary material for: Clarithromycin inhibits autophagy in colorectal cancer by regulating the hERG1 potassium channel interaction with PI3K
Source: Cell Death Dis. 2020 Mar 2;11(3):161. doi: 10.1038/s41419-020-2349-8 (PMC7052256; doi:10.1038/s41419-020-2349-8)
Supplement: Supplementary file 1 — Supplementary figure and table legends [file 41419_2020_2349_MOESM1_ESM.docx]

**Supplementary Materials**

**Fig. S1.** **Effects of Clarithromycin on cell viability of HCT116 cells.**

Cell viability of HCT116 cells was assessed after 24 hours of treatment with Clarithromycin (Cla) (range 0 - 200 µM), with the Trypan Blue exclusion test. Data are presented as mean ± SEM of four independent experiments, each carried out in triplicate. Statistical significance reported for comparison of Cla-treated cells vs control cells, was assessed with a one-way ANOVA; **, *P* < 0.01 and ***, *P* < 0.001.

**Fig. S2.** **Vacuoles formation in three human colorectal cancer (CRC) cell lines treated with a high dose of Clarithromycin.**

(Left Panel) Effect of Cla (80 and 160 µM) on size and number of vacuoles were respectively quantified by measuring the diameters of all vacuoles and counting all vacuoles in 10-12 HCT116 cells treated for 24 hours, from three independent experiments. For the analysis were evaluated only HCT116 cells, treated with Cla and stained with May-Grümwald and Giemsa, which presented visible vacuoles. Data are reported as mean ± SEM of three independent experiments. (Middle panels) HCT116, HT29 and LS174T cells were incubated for 24 hours with Cla at their relative 2 X IC_50_ values, indicated in the lower left corner of each picture, then stained with May-Grümwald and Giemsa in order to evaluate vacuoles formation. Representative images of HCT116 cells stained with May-Grümwald and Giemsa, are reported (original magnification, X400; scale bar, 100 µm). (Right panel) Percentages of cells with vacuoles are reported in the bar graph as mean ± SEM (*n* = 3). Statistical significance reported for comparison of Cla-treated cells vs control cells, was assessed with a one-way ANOVA; ***, *P* < 0.001.

**Fig. S3.** **Clarithromycin-induced vacuoles formation in HCT116 silenced for *hERG1* or cotreated with E4031.**

(**a – c**) HCT116 cells were treated with Lipofectamine 2000 and silenced with specific α-*hERG1* siRNAs (hERG1-KD) or with siRNA negative control (siRNA neg). (**a**) Knockdown of hERG1 in HCT116 was confirmed by WB. (**b-c**) Twenty-four hours after siRNA transfection, the medium was changed and cells were treated with Cla (80 µM). HCT116 wild type (WT) cells were also treated with Cla (80 µM), alone or in combination with E4031 (40 µM). After 24h of incubation, cells were harvested and accumulation of acidic vesicular organelles (AVOs) (**b**) and formation of cytoplasmic vacuoles (**c**) were investigated, respectively by Acridine Orange (AO) and May-Grümwald and Giemsa. (**b**) Representative dot plots of AO staining are shown taken from three independent experiments. Quantification of AVOs is expressed as the percentage of AVOs-positive cells (indicated on the representative dot plots). (**c**) Representative images of cells stained with May-Grümwald and Giemsa, are reported (original magnification, X400; scale bar, 50 µm).

**Fig. S4. Confocal imaging of hERG1 mutants and hERG1-WT-transfected HEK293 cells, treated with fluorescently labelled Clarithromycin.**

Single plane confocal microscopy images of hERG1 mutants and hERG1-WT-transfected HEK293 cells treated for 30 minutes with 10 µM 11-NBD-Cla. Bar graph shows mean 11-NBD-Cla mean fluorescence intensity, normalized on selected cell area. The results are representative of three independent experiments (*n* = 21 number of cells analyzed for mutant) and presented as means ± SEM. Scale bar, 20 µm. Statistical significance was assessed by one-way ANOVA. *, *P* < 0.05 and ***, *P* < 0.001.

**Fig. S5. Membrane abundance of hERG1 constructs measured in HCT116 cells.**

Expression of the indicated hERG1 constructs transfected in HCT116 cells, measured by flow cytometry and expressed as mean fluorescence intensity (MFI). The mean fluorescence intensity of mock-transfected HCT116 cells was set as 1.

**Fig S6. LC3 and phospho-ERK expression analysis in hERG1-G628S HEK mutants.**

(**a**) WB analysis of LC3 expression in HCT116 cells expressing hERG1-G628S mutant and treated with control or Cla (80 and 160 µM) for 4 48 hours (*n* = 1). (**b**) The densitometric results of phospho-ERK1/2^Thr202/Tyr204^ levels in HCT116 cells expressing hERG1 (hERG1-WT), or hERG1-K525C, or hERG1-R531C, or hERG1-G628S and treated with Cla (80 and 160 µM) for 4 hours. Representative WBs are reported in Fig. 4d (*n* = 2).

**Fig. S7.** **Expression of** **the** **potassium channel KCa3.1 assessed by western blot analysis.**

WB analysis of KCa3.1 protein expression in HCT116 cells treated with Cla (80 and 160 µM) for 4, 24 and 48 hours (*n* = 1).

**Fig. S8. Evaluation of the effect of Clarithromycin on autophagy in HCT116 p53^-/-^ cells.**

Quantification of AVOs in HCT116 p53^-/-^ cells treated with Cla (80 µM) for 24 hours and analyzed as in fig. S3. Representative dot plots of AO staining are shown taken from three independent experiments. Quantification of AVOs is expressed as the percentage of AVOs-positive cells (indicated on the representative dot plots). Data are reported as mean ± SEM of three independent experiments.

**Fig. S9.** **Effects of Clarithromycin on proliferation of LS174T cells.**

Effects of Cla on proliferation (expressed as the number of alive, Trypan Blue-negative, cells) of LS174T cells, after a single (arrow; upper panels) and a double treatment (arrows; lower panels). Cla was added at the indicated concentrations. Data presented as mean ± SEM of three independent experiments, each carried out in triplicate. *, *P* < 0.05*,* for the indicated comparison (one-way ANOVA). Other results of statistical analysis assessed by one-way ANOVA: after 24 hours of treatment: Cla 160 vs Control, *P* < 0.05. After 48 hours: Cla 160 vs Control, *P* < 0.05. After 72 hours of double treatment (lower panel): Cla 80 vs Control, *P* < 0.05; Cla 160 vs Control, *P* < 0.001.

**Fig. S10. Effects of Clarithromycin on cell cycle distribution and apoptosis of HCT116 cells, after 24 hours of incubation.**

(**a** - **b**) HCT116 cells were treated with Cla (80 and 160 µM). After 24 hours of treatment, cells were harvested and stained for flow cytometric analysis of cell cycle distribution (**a**) and apoptosis (**b**). Representative histograms of cell cycle analysis and relative percentages of gated cells at sub-G_1_, G_1_/G_0_, S and G_2_/M phases are reported in (**a**). Representative dot plots of Annexin-V/PI analysis and relative percentages of gated cells for alive (annexin V-negative and PI-negative), necrotic (annexin V-negative and PI-positive), early apoptotic (annexin V-positive and PI-negative) and late apoptotic (annexin V-positive and PI-positive) HCT116, are reported in (**b**) (*n* = 4) Statistical significance was assessed with a one-way ANOVA test for **b**; *, *P* < 0.05.

**Fig. S11. Effects of Clarithromycin on cell viability of HEK293 cells overexpressing hERG1.**

Cell viability of mock-transfected HEK293 (HEK293 MOCK) and hERG1-transfected HEK293 (HEK293 hERG1) cells assessed after 24 hours of treatment with Cla (range 0-200 µM), with the Trypan Blue exclusion test. Data presented as mean ± SEM of four independent experiments, each carried out in triplicate. Statistical significance reported for comparison of Cla-treated cells vs control cells, was assessed with a one-way ANOVA; **, *P* < 0.01 and ***, *P* < 0.001.

**Fig. S12. Evaluation of the effect of Clarithromycin on apoptosis induction in HCT116 silenced for *hERG1* and in HCT116 p53^-/-^ cells.**

(**a**) HCT116 cells treated with Lipofectamine 2000 and silenced with specific anti-hERG1 siRNAs (hERG1-KD) or with siRNA negative control (siRNA neg). Twenty-four hours after siRNA transfection, cells were treated with control or Cla (160 µM). After 48h of treatment, cells were collected and stained for flow cytometric analysis and apoptotic cell death. Representative dot plots of three independent experiments, are reported. (**b**) HCT116 p53^-/-^ cells were treated with control or Cla (160 µM). After 48h of treatment, cells were collected and stained for flow cytometric analysis of apoptotic cell death. Representative dot plots of three independent experiments, are reported.

**Fig. S13.** **Effects of Irinotecan and 5-Fluorouracil, alone or in combination with Cla, on cell viability of HCT116 cells.**

(**a**, upper panels) Effects of irinotecan (CPT-11) and 5-Fluoruracil (5-FU) on HCT116 cells viability after 24 hours of treatment. Data are given as the number of Trypan Blue negative cells. Data are reported as means ± SEM of four independent experiments, each carried out in triplicate. **, P < 0.05, **, P < 0.01, ***, P < 0.001*, for comparison of drug-treated cells vs control cells (one-way ANOVA). (**a,** lower panels) Dose-response curve of CPT-11 and 5-FU (right panel) effects on HCT116 viability. Curves reported were obtained by non-linear curve fitting (Hill1) by OriginPro 8 software. (**b**) Cell viability after 24 hours of treatment with CPT-11 (left panel) and 5-FU (right panel) in combination with Cla. Drugs were used at their relative IC_50_ and IC_25_ doses. Data are means ± SEM of three independent experiments, each carried out in triplicate. **, P < 0.05, **, P < 0.01, ***, P < 0.001,* for the indicated comparison (one-way ANOVA).

**Fig. S14. Evaluation of the synergic effect of 5-Florouracil and Clarithromycin on apoptosis induction in HCT116 cells.**

HCT116 cells were treated with 5-FU (13.7 µM) alone or in combination with Cla (80 µM). After 24 and 48 hours of treatment, cells were collected and stained for flow cytometric analysis and apoptotic cell death. Representative dot plots of three independent experiments, are reported.

**Fig. S15. Twenty-four hours’ effects of 5-Fluoruracil and Clarithromycin on HCT116 spheroids volume.**

Dose-response curve of 5-Fluoruracil (5-FU) (left panel) and Clarithromycin (Cla) (right panel) effects on HCT116 spheroids volume. Spheroid volumes were measured by Matlab and curves reported were obtained by non-linear curve fitting (Hill1) by OriginPro 8 software.

**Fig. S16. Evaluation of the synergic effect of 5-Florouracil and Clarithromycin on cell death in HCT116 cells cultured as spheroids.**

Three-dimensional (3D) spheroids were treated with 5-FU or Cla, alone and in combination, at their relative IC_50_ and IC_25_ values obtained for HCT116 cultured in two-dimensional cultures (5-FU, IC_25_: 2.2 µM and IC_50_: 13.7 µM; Cla, IC_25_: 40 µM and IC_50_: 80 µM). After 120 hours of treatment the cell viability of spheroids were assessed, by using a live/dead staining with Calcein AM (2 µg/mL) and PI (10 µg/mL) and incubated for 20 minutes at 37°C^55^. Representative Images of the cell viability assay of spheroids assessed after 120 hours of treatment, by staining with PI and calcein AM. Viable cells appear as green, while nonviable cells appear as red. Scale bars, 100 μm (n =2 independent experiments each conducted with 6 sample for each concentration).

**Fig. S17.** **Expression of** **hERG1 protein in human CRC cell lines assessed by western blot analysis.**

Expression of hERG1 protein in the indicated cell lines evaluated by WB. Representative blots of three independent experiments, are reported.

**Fig. S18.** **Percentage of hERG1 positive samples.**

In the graph bars are reported the percentage of positive samples for hERG1 (≥50% of positive cells; immunohistochemical score described in^45^ in normal (0%, 0/13), stage I/II (29%, 36/124), stage III (29%, 26/89) and stage IV (67%, 79/118) samples.

A retrospective study was conducted on a cohort of 344 patients with colorectal adenocarcinoma. Patients were selected by the medical oncologists of Azienda Ospedaliero Universitaria, Careggi, Florence, of the Spedali Civili Hospital, Brescia and Campus Biomedico University of Rome. Healthy mucosa (*n* = 13), stage I/II (*n* = 124), III (*n* = 89) and IV (*n* = 118) patients were treated with surgery in all institutions. The study was carried out with approval of the local Ethical Committee. 331 formalin-fixed, paraffin-embedded CRC samples stage I/II (*n* = 124), stage III (*n* = 89) and stage IV (*n* = 118) and 13 healthy mucosa CR belonging to CRC patients samples were analysed for the expression of hERG1. After dewaxing and rehydrating the sections, endogenous peroxidases were blocked with a 1% H_2_O_2_ solution in phosphate-buffered saline (PBS). Subsequently, antigen retrieval was performed by treatment with proteinase K (5 µg/ml) in PBS at 37°C for 5 minutes. The following antibody was used: anti-hERG1 monoclonal antibody (MCK Therapeutics; 0,005 µg/µl. Incubation with the primary antibody was carried out overnight at 4°C. Immunostaining was performed with a commercially available kit (PicTure max kit; Invitrogen) according to the manufacturer's instructions. Samples were evaluated by two independent investigators (GP and JI). The expression of hERG1 was evaluated by an immunohistochemical score obtained through the percentage of immunoreactive cells (quantity score) (described in^45^). No staining was scored as 0, 1–49% of stained cells was scored as 1, 50-100% as 2.

**Table S1. List of antibodies used in the study.**

**Table S2. List of chemicals used in the study.**

**Table S3. Results of statistical analysis relative to Figs. 5a and 7a**
