# Supplementary figures and images for: Clarithromycin inhibits autophagy in colorectal cancer by regulating the hERG1 potassium channel interaction with PI3K
Source: Cell Death Dis. 2020 Mar 2;11(3):161. doi: 10.1038/s41419-020-2349-8 (PMC7052256; doi:10.1038/s41419-020-2349-8)

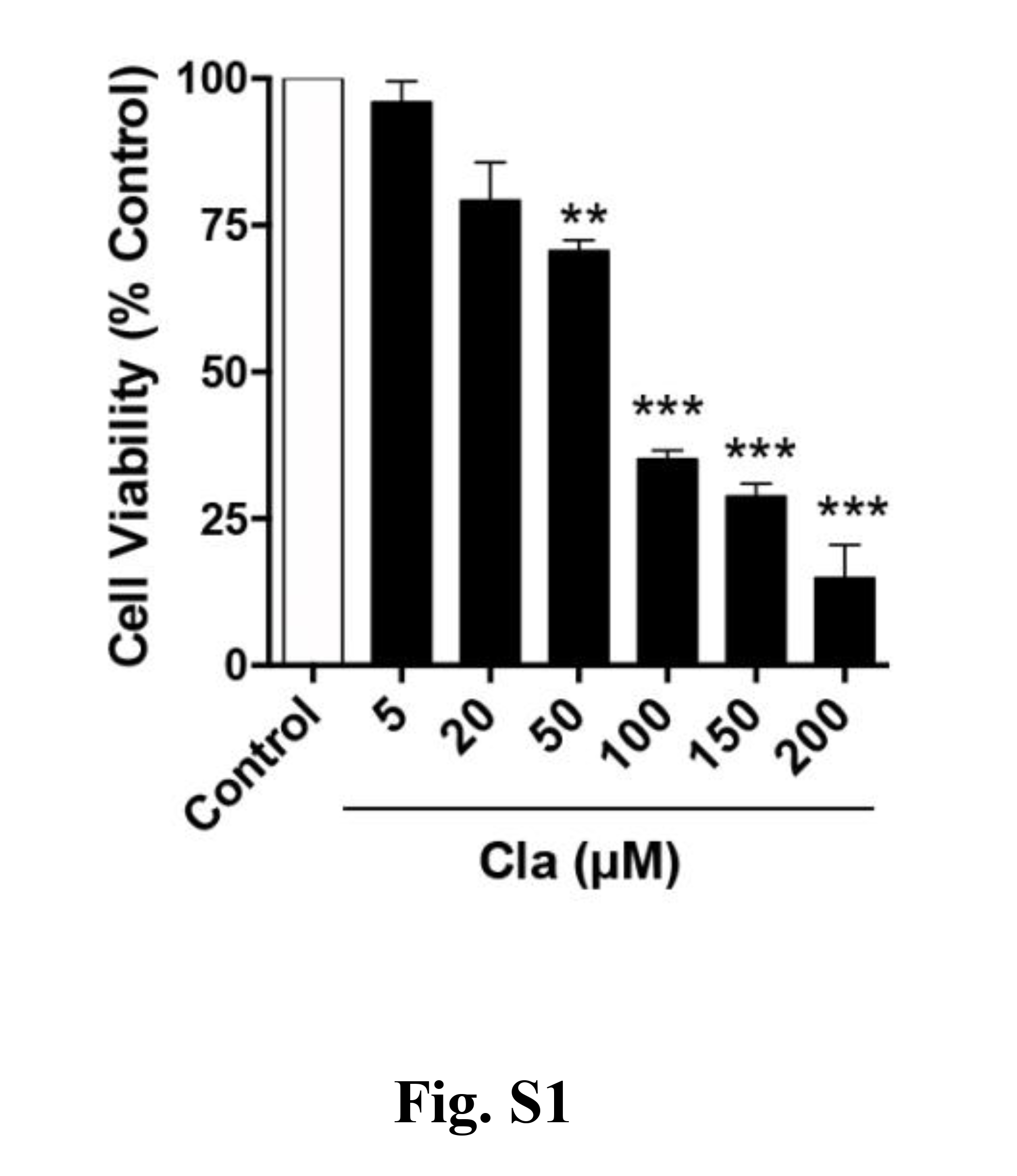

Supplement: Supplementary file 2 — Supplementary Figure S1 [file 41419_2020_2349_MOESM2_ESM.tif]

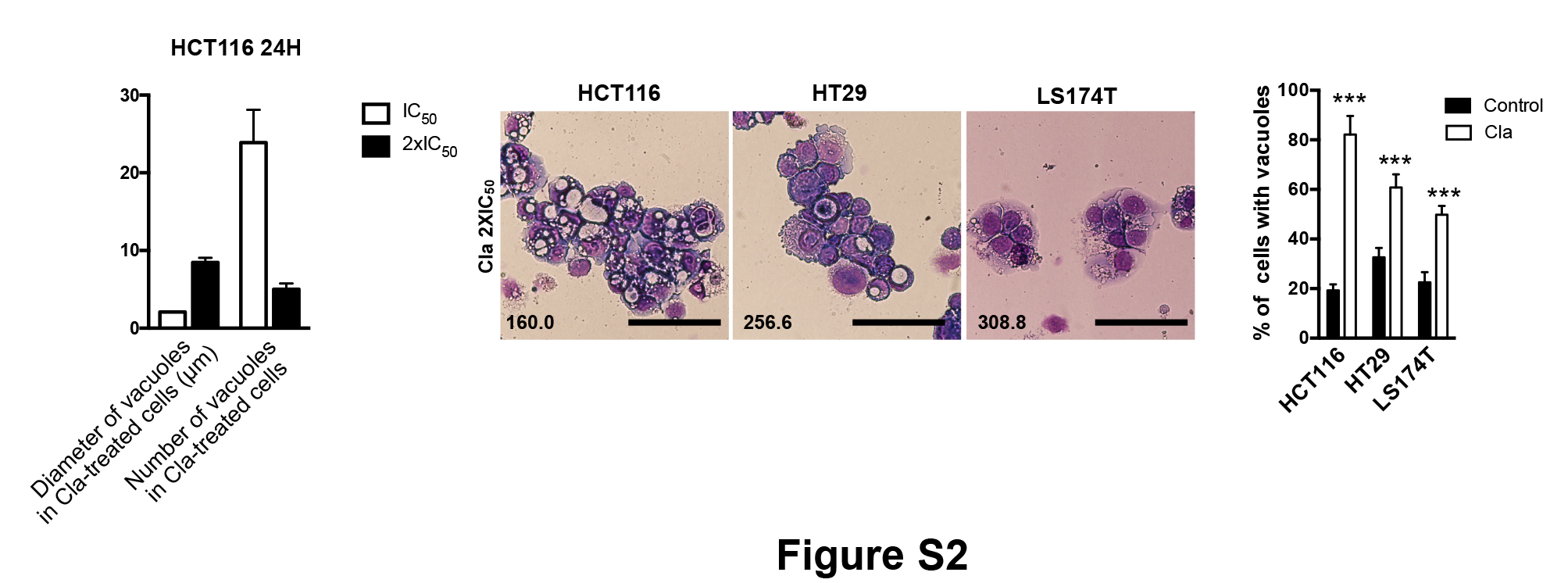

Supplement: Supplementary file 3 — Supplementary Figure S2 [file 41419_2020_2349_MOESM3_ESM.tif]

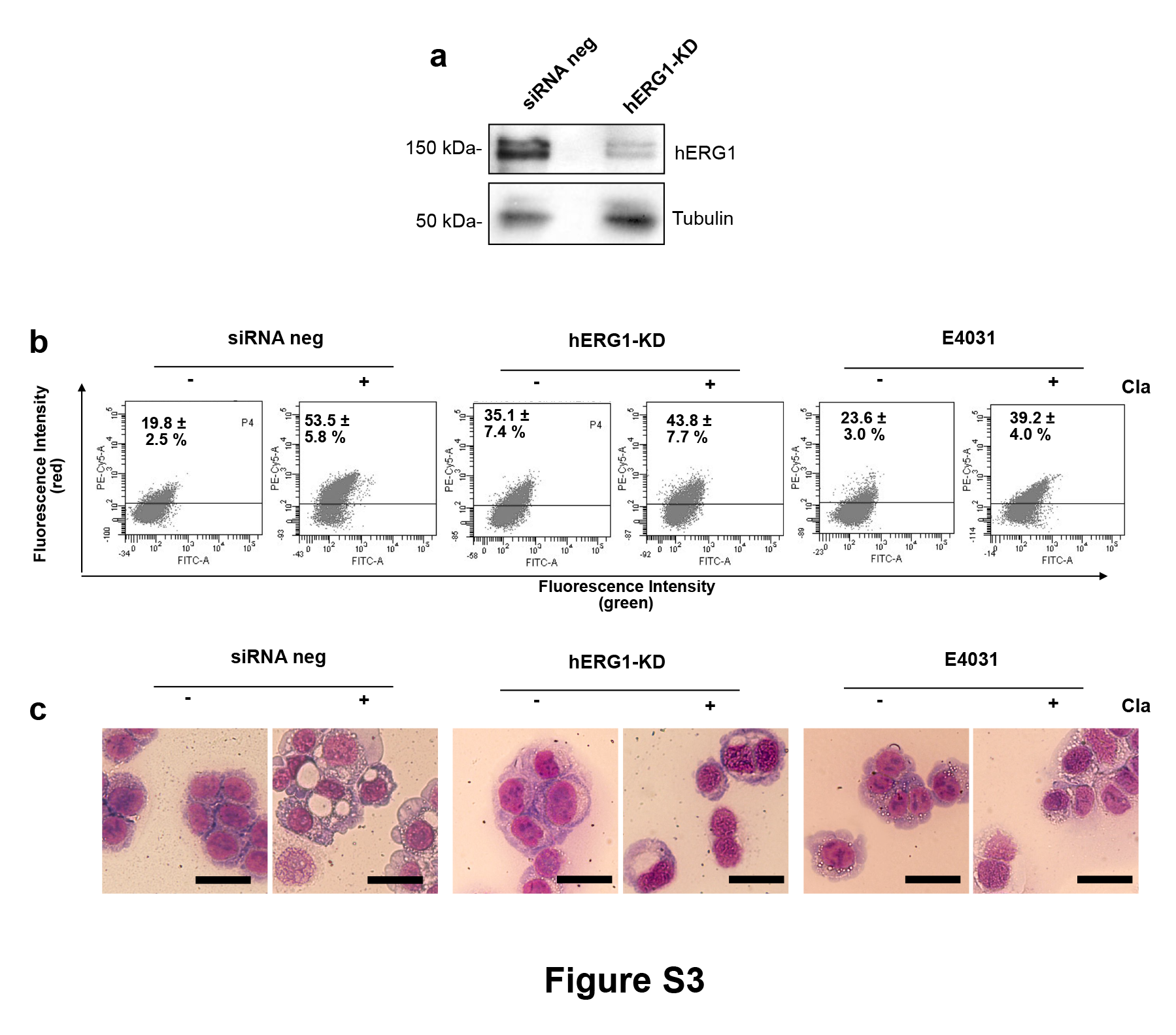

Supplement: Supplementary file 4 — Supplementary Figure S3 [file 41419_2020_2349_MOESM4_ESM.tif]

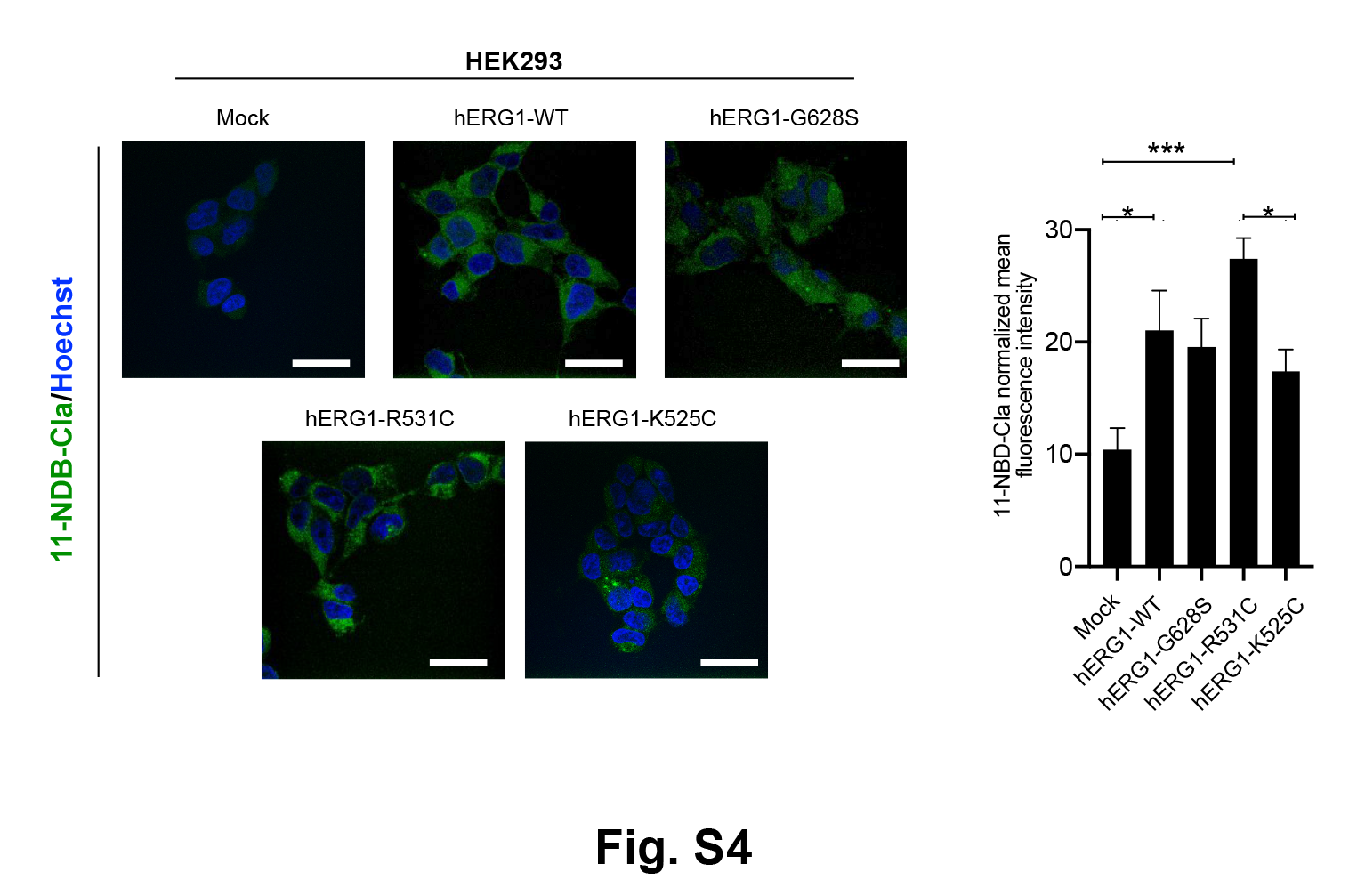

Supplement: Supplementary file 5 — Supplementary Figure S4 [file 41419_2020_2349_MOESM5_ESM.tif]

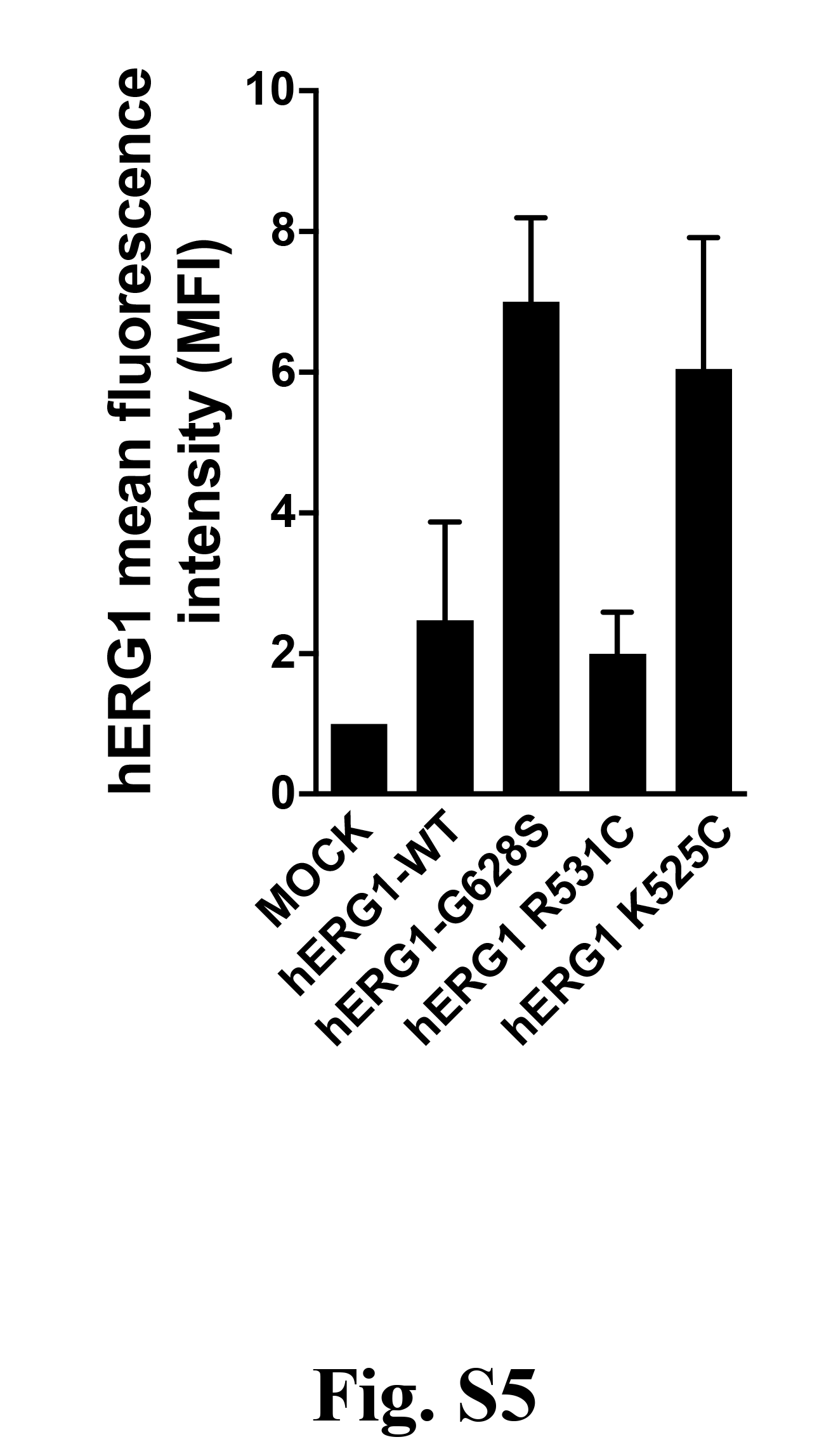

Supplement: Supplementary file 6 — Supplementary Figure S5 [file 41419_2020_2349_MOESM6_ESM.tif]

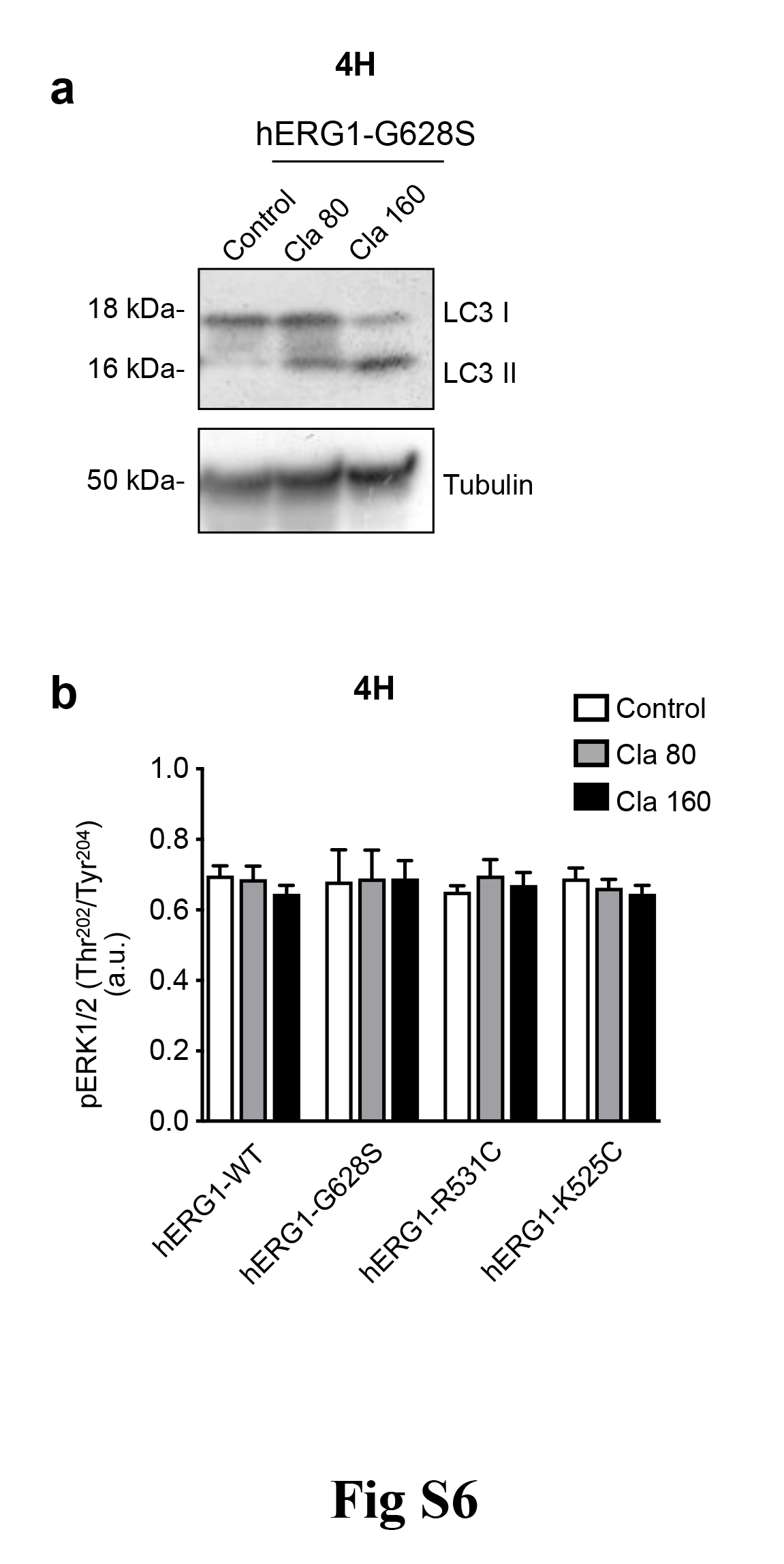

Supplement: Supplementary file 7 — Supplementary Figure S6 [file 41419_2020_2349_MOESM7_ESM.tif]

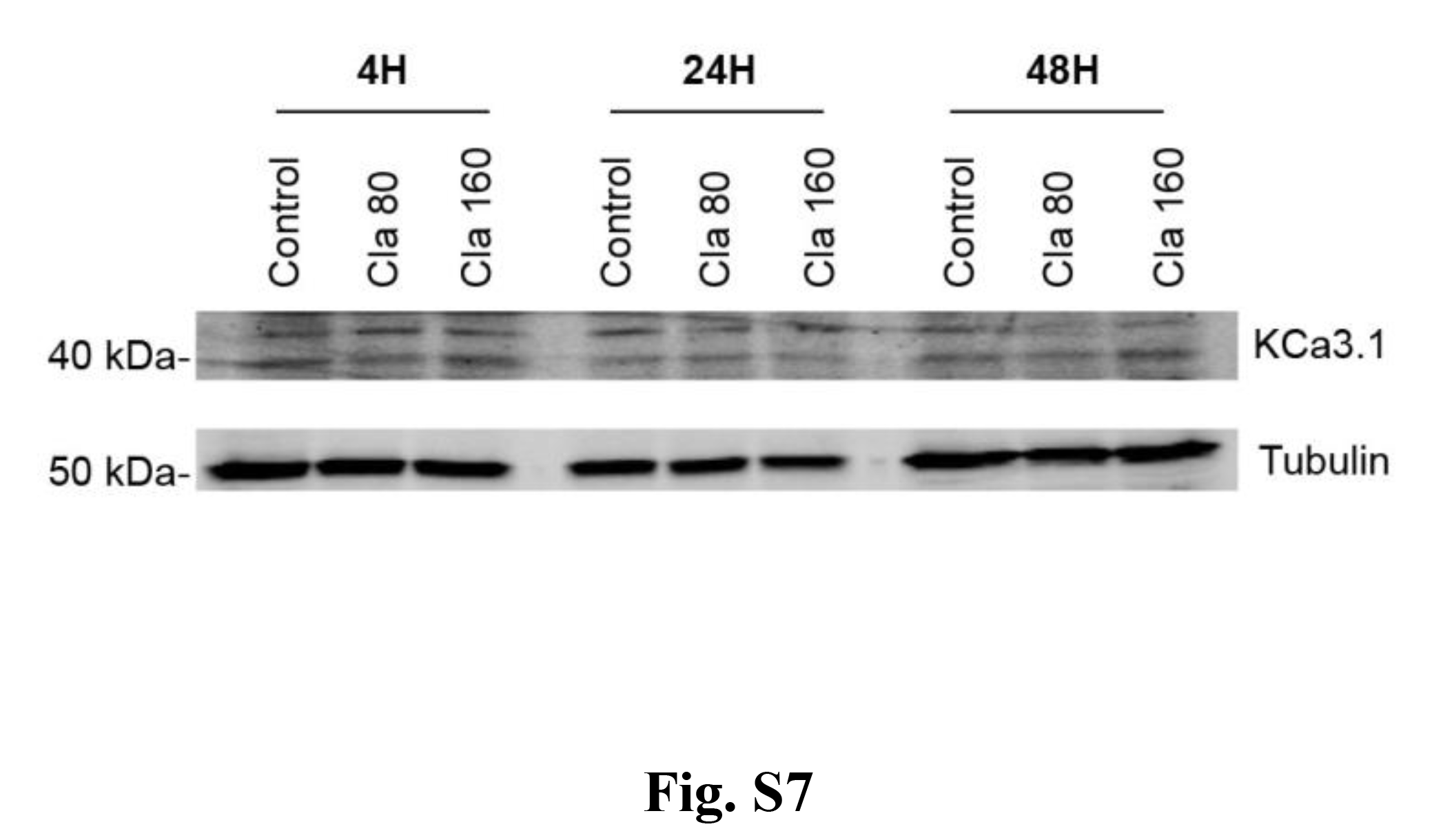

Supplement: Supplementary file 8 — Supplementary Figure S7 [file 41419_2020_2349_MOESM8_ESM.tif]

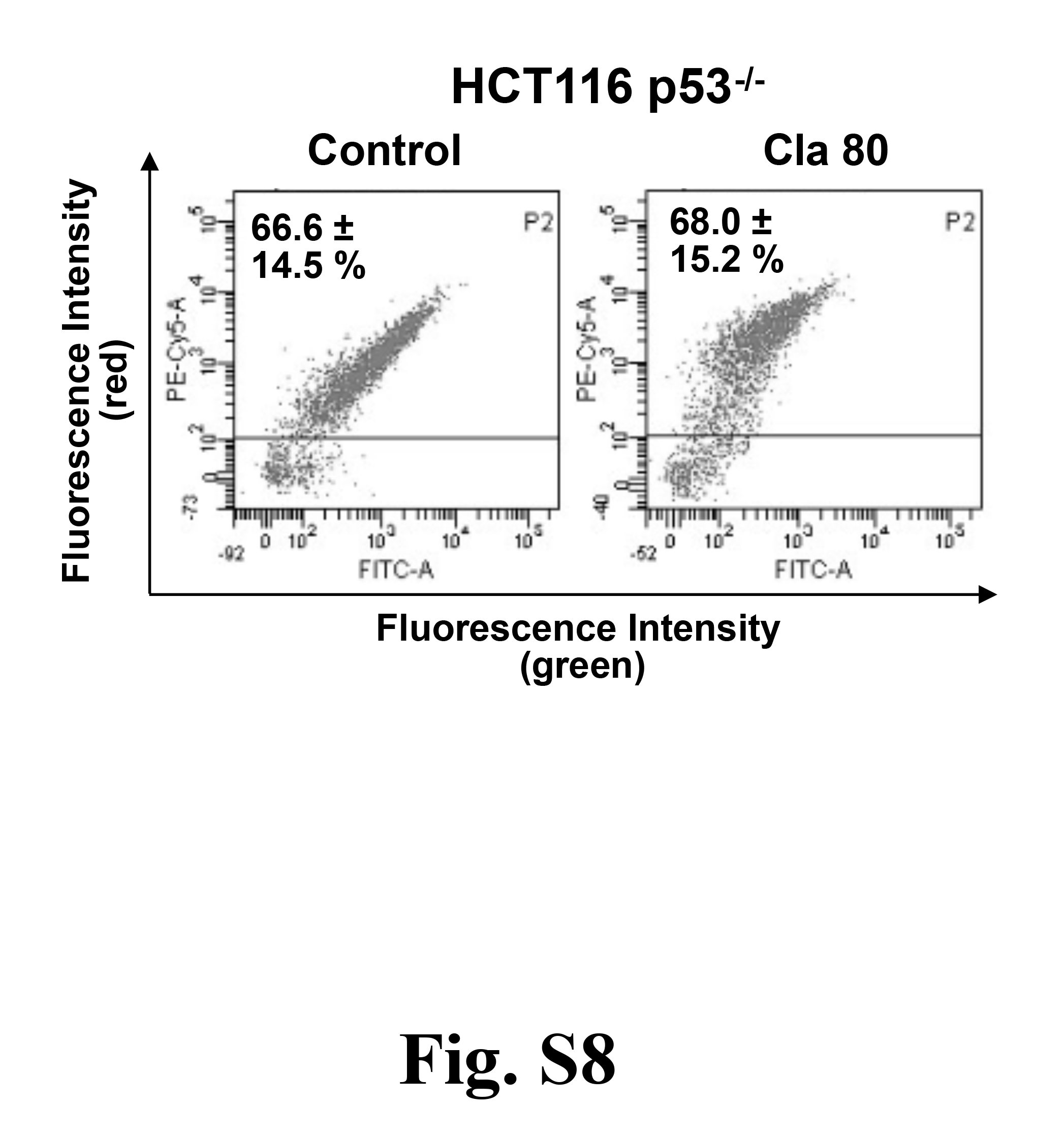

Supplement: Supplementary file 9 — Supplementary Figure S8 [file 41419_2020_2349_MOESM9_ESM.tif]

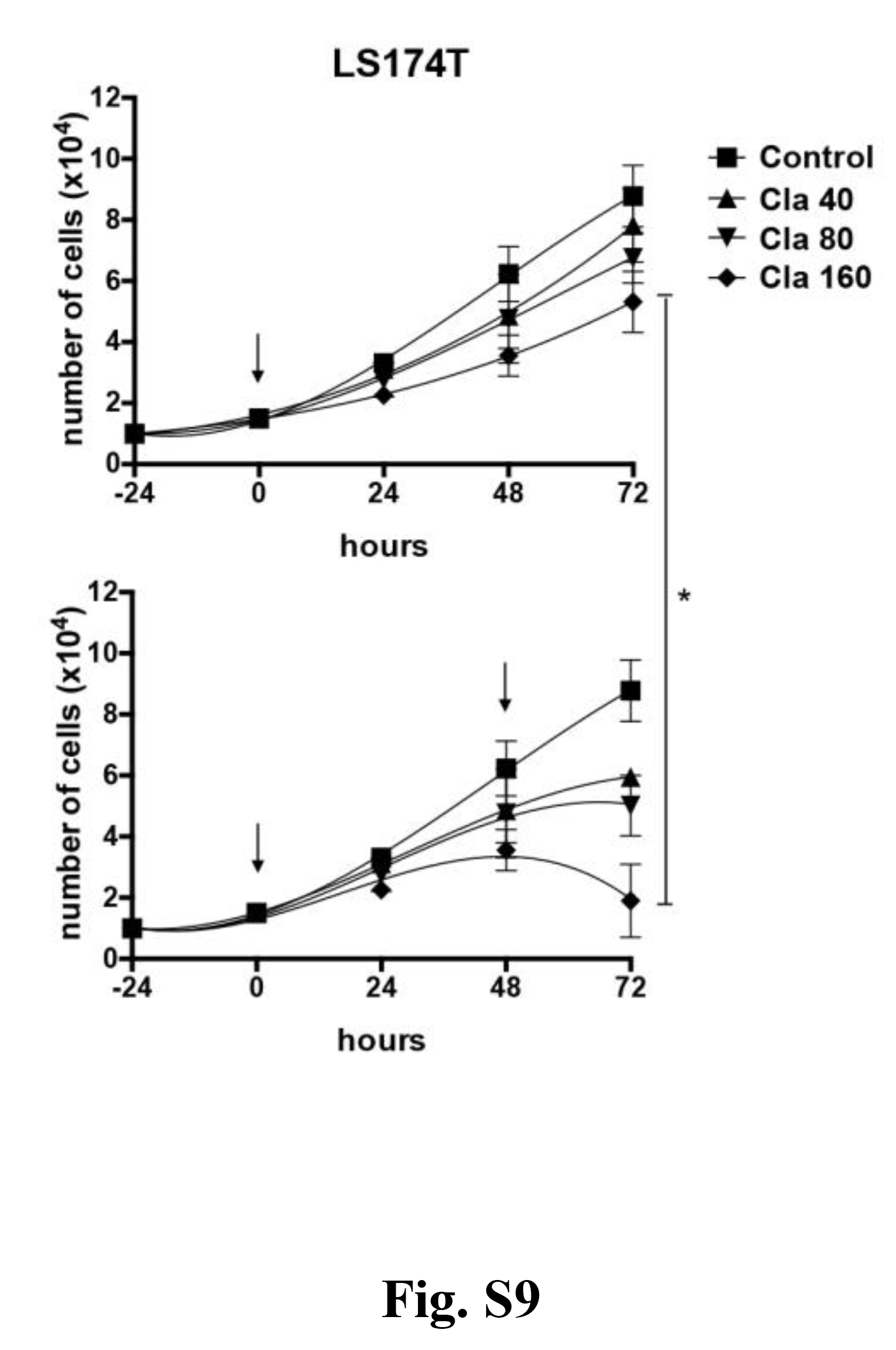

Supplement: Supplementary file 10 — Supplementary Figure S9 [file 41419_2020_2349_MOESM10_ESM.tif]

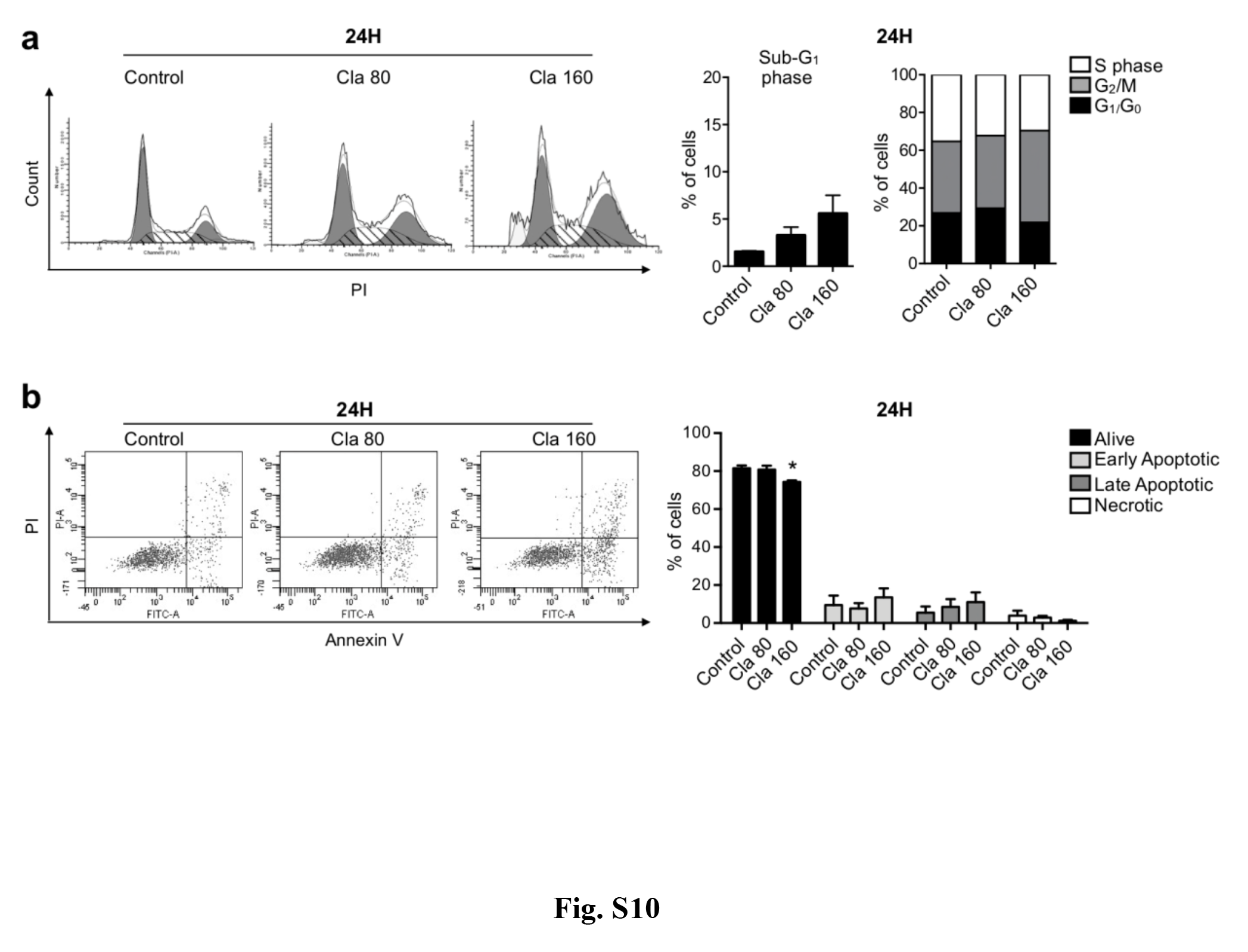

Supplement: Supplementary file 11 — Supplementary Figure S10 [file 41419_2020_2349_MOESM11_ESM.tif]

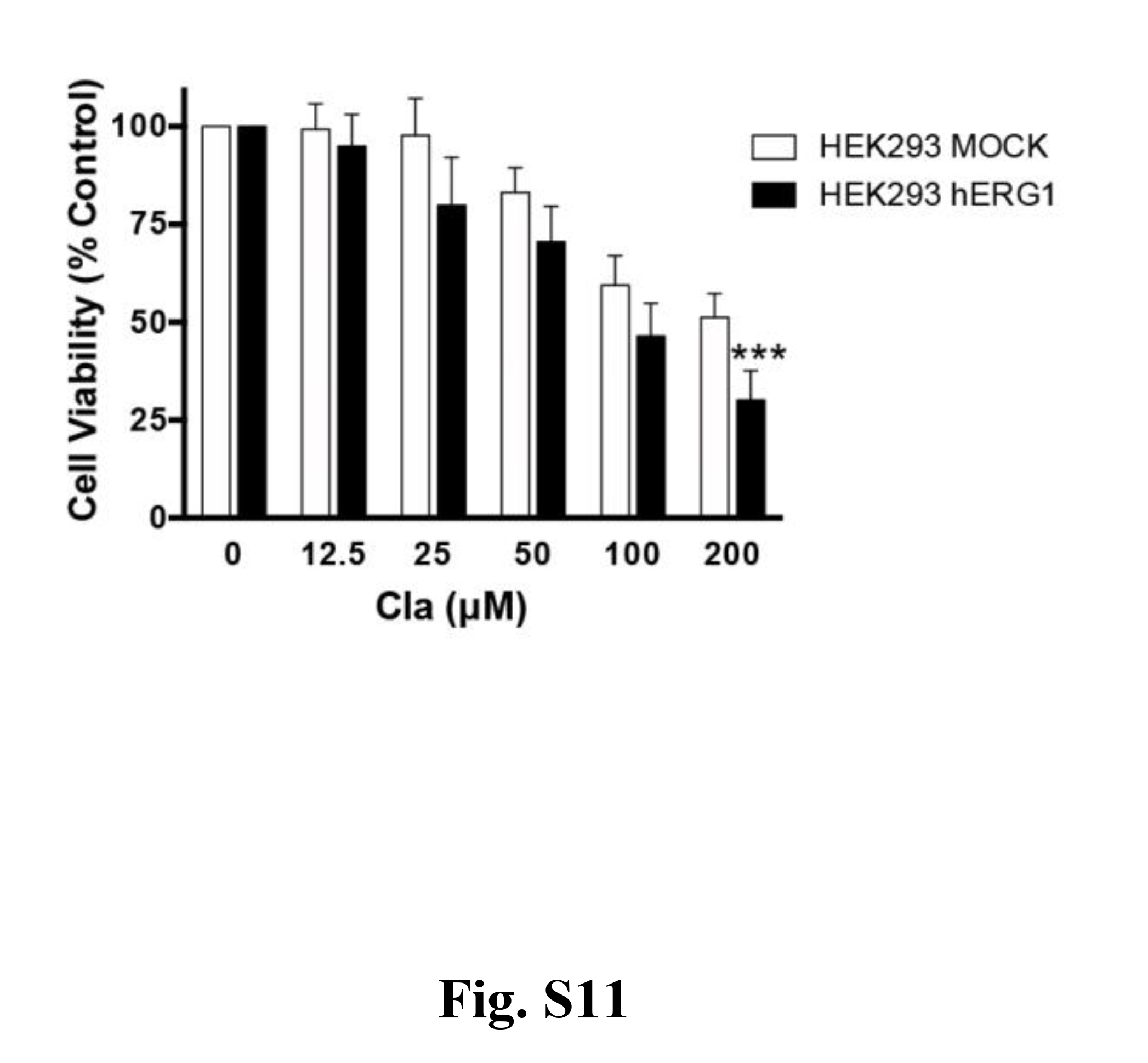

Supplement: Supplementary file 12 — Supplementary Figure S11 [file 41419_2020_2349_MOESM12_ESM.tif]

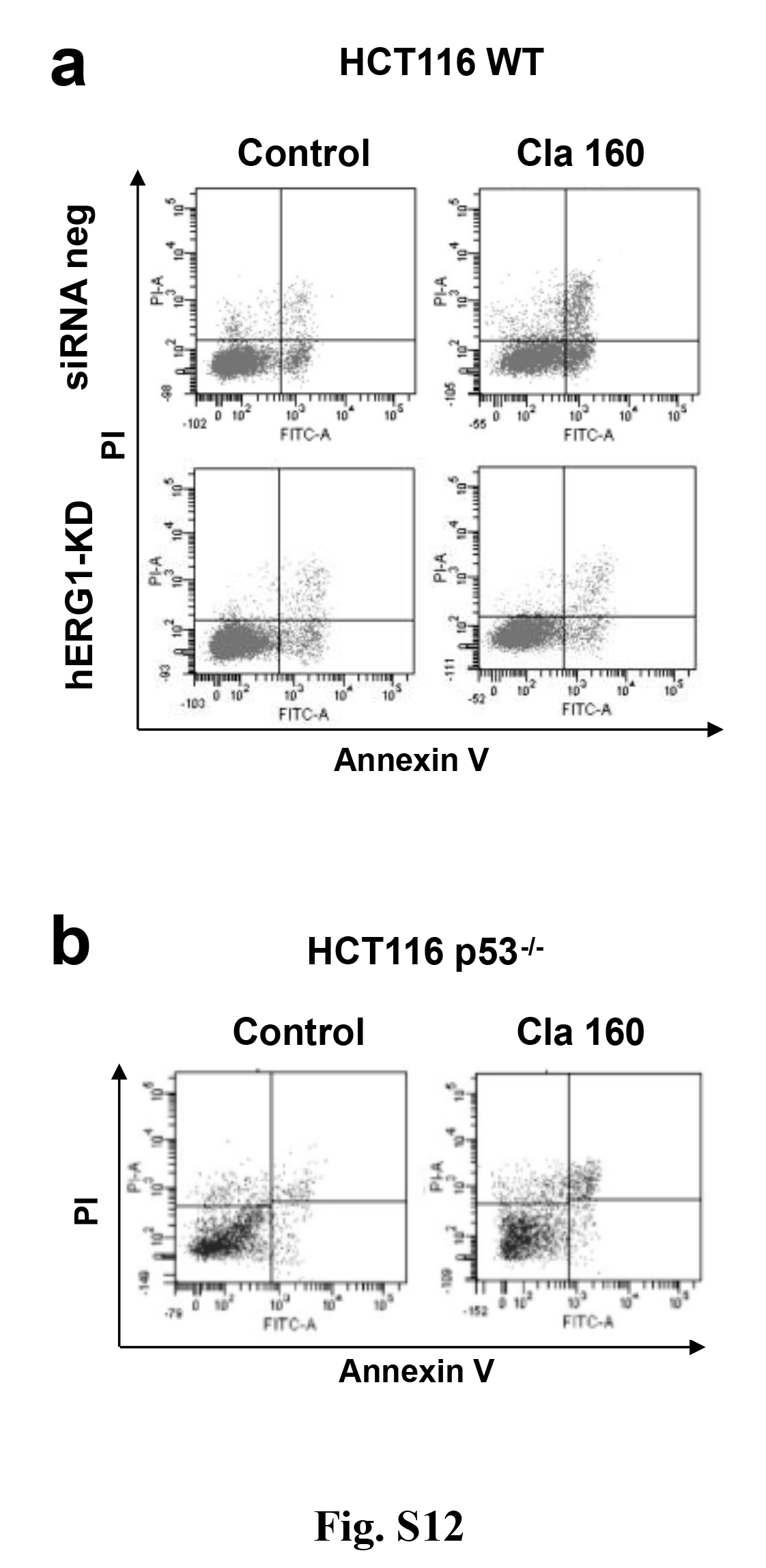

Supplement: Supplementary file 13 — Supplementary Figure S12 [file 41419_2020_2349_MOESM13_ESM.tif]

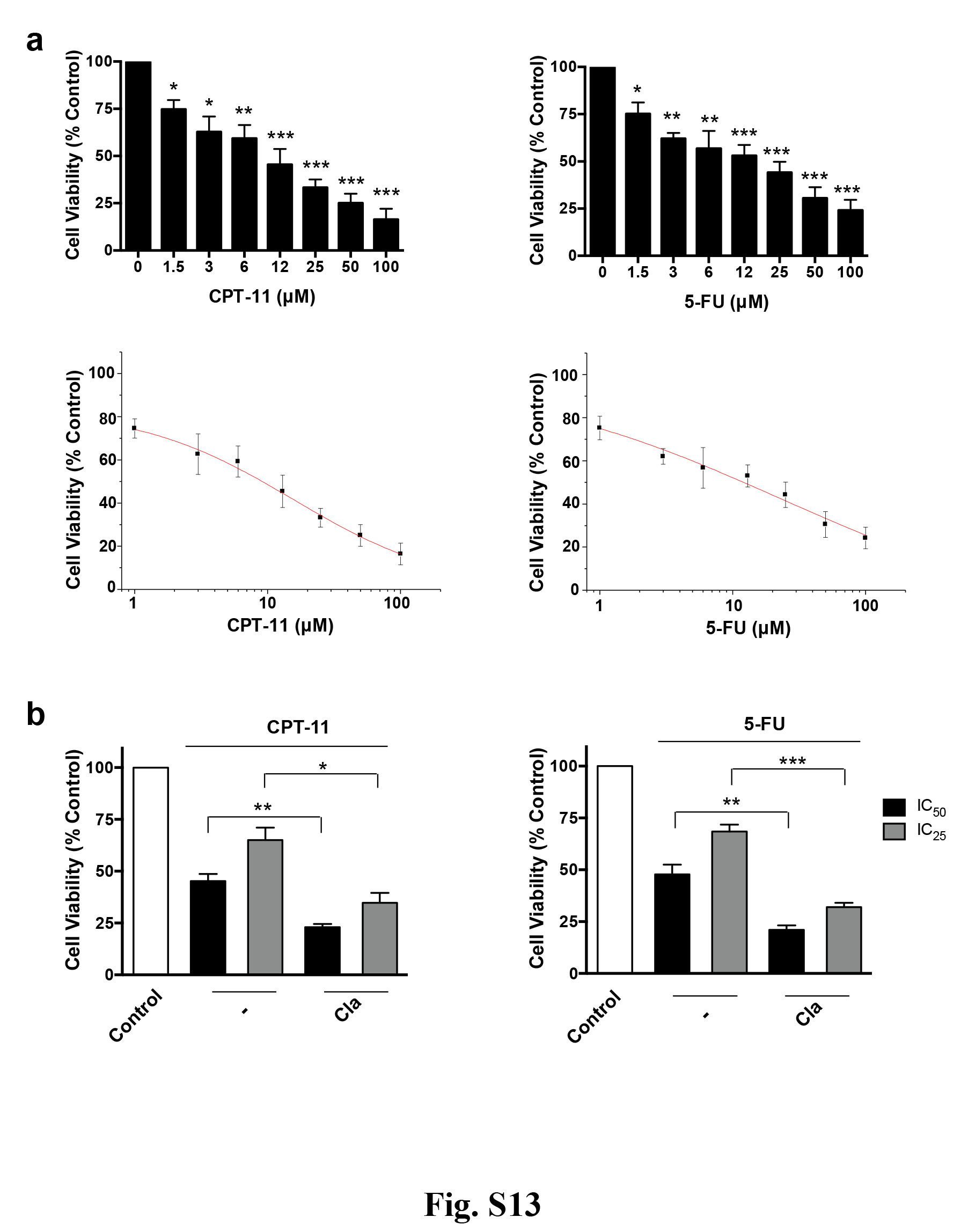

Supplement: Supplementary file 14 — Supplementary Figure S13 [file 41419_2020_2349_MOESM14_ESM.tif]

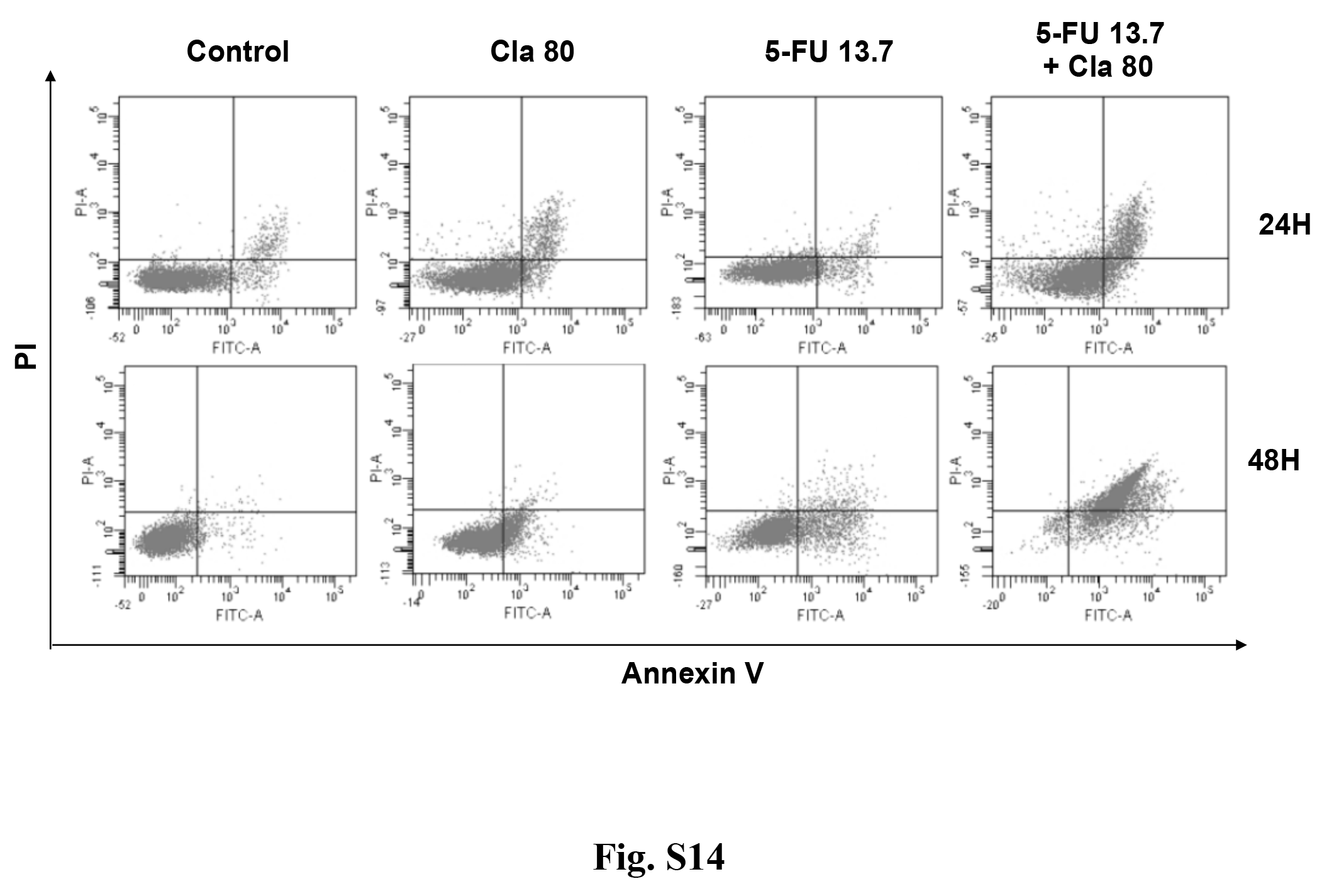

Supplement: Supplementary file 15 — Supplementary Figure S14 [file 41419_2020_2349_MOESM15_ESM.tif]

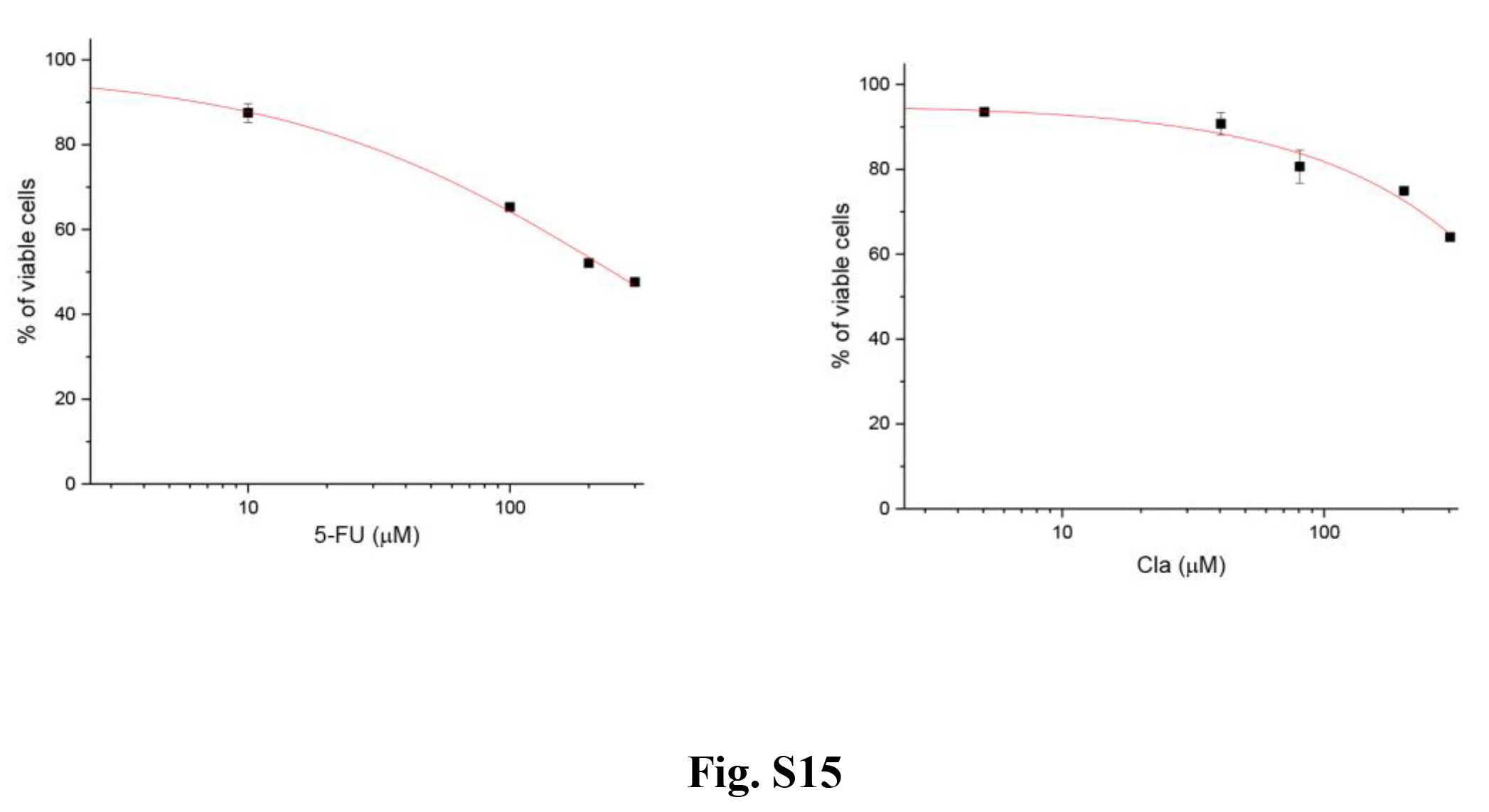

Supplement: Supplementary file 16 — Supplementary Figure S15 [file 41419_2020_2349_MOESM16_ESM.tif]

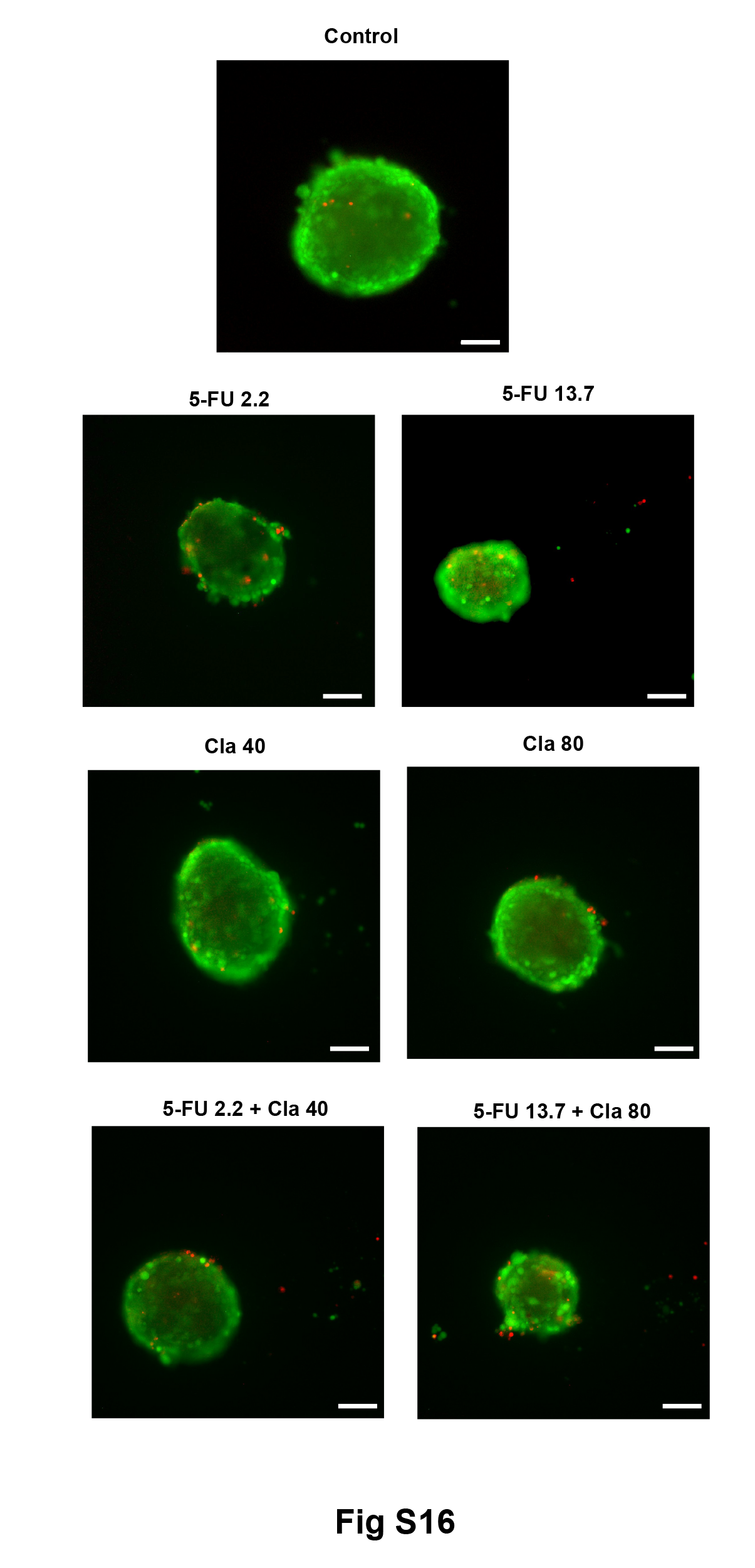

Supplement: Supplementary file 17 — Supplementary Figure S16 [file 41419_2020_2349_MOESM17_ESM.tif]

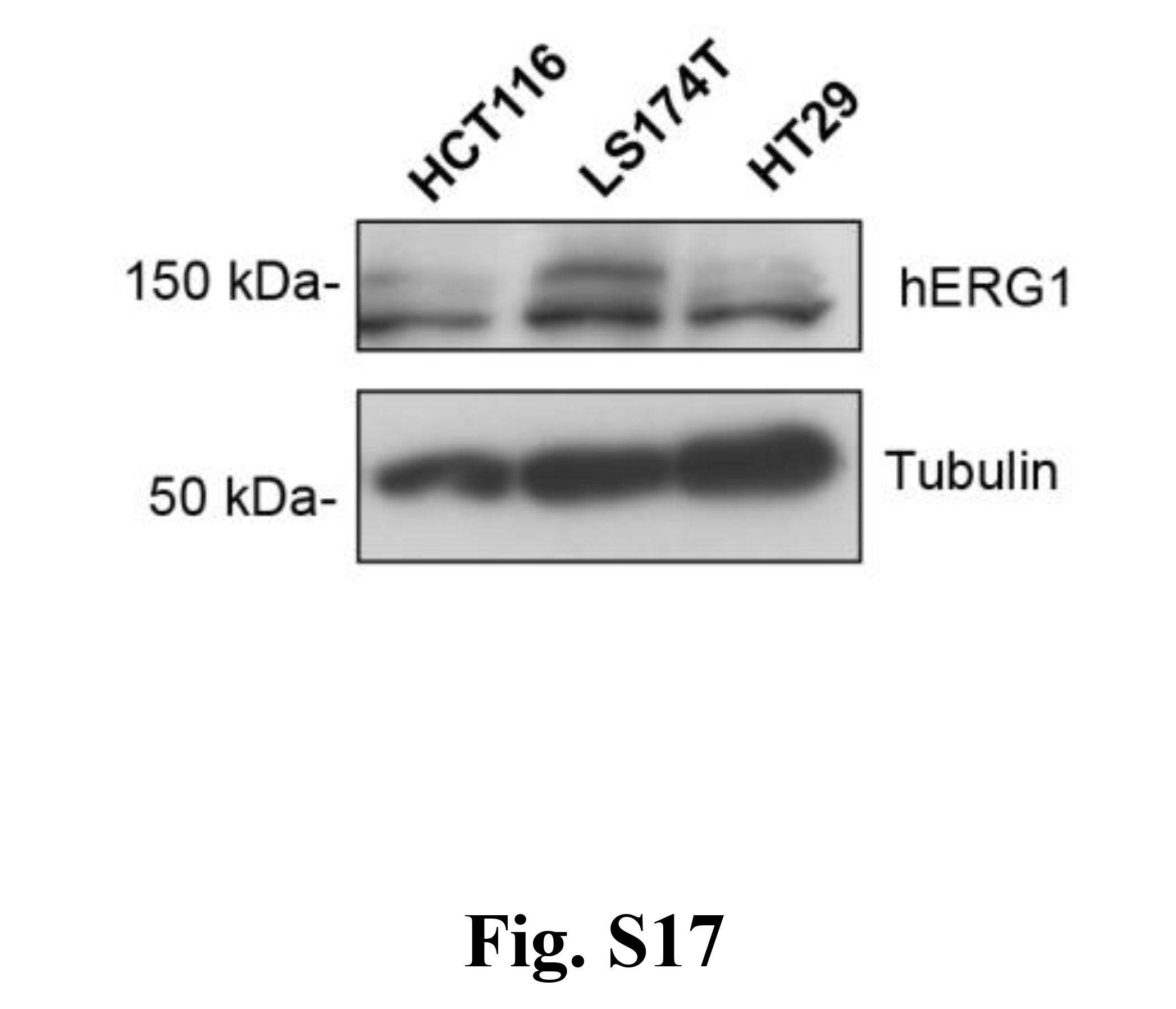

Supplement: Supplementary file 18 — Supplementary Figure S17 [file 41419_2020_2349_MOESM18_ESM.tif]

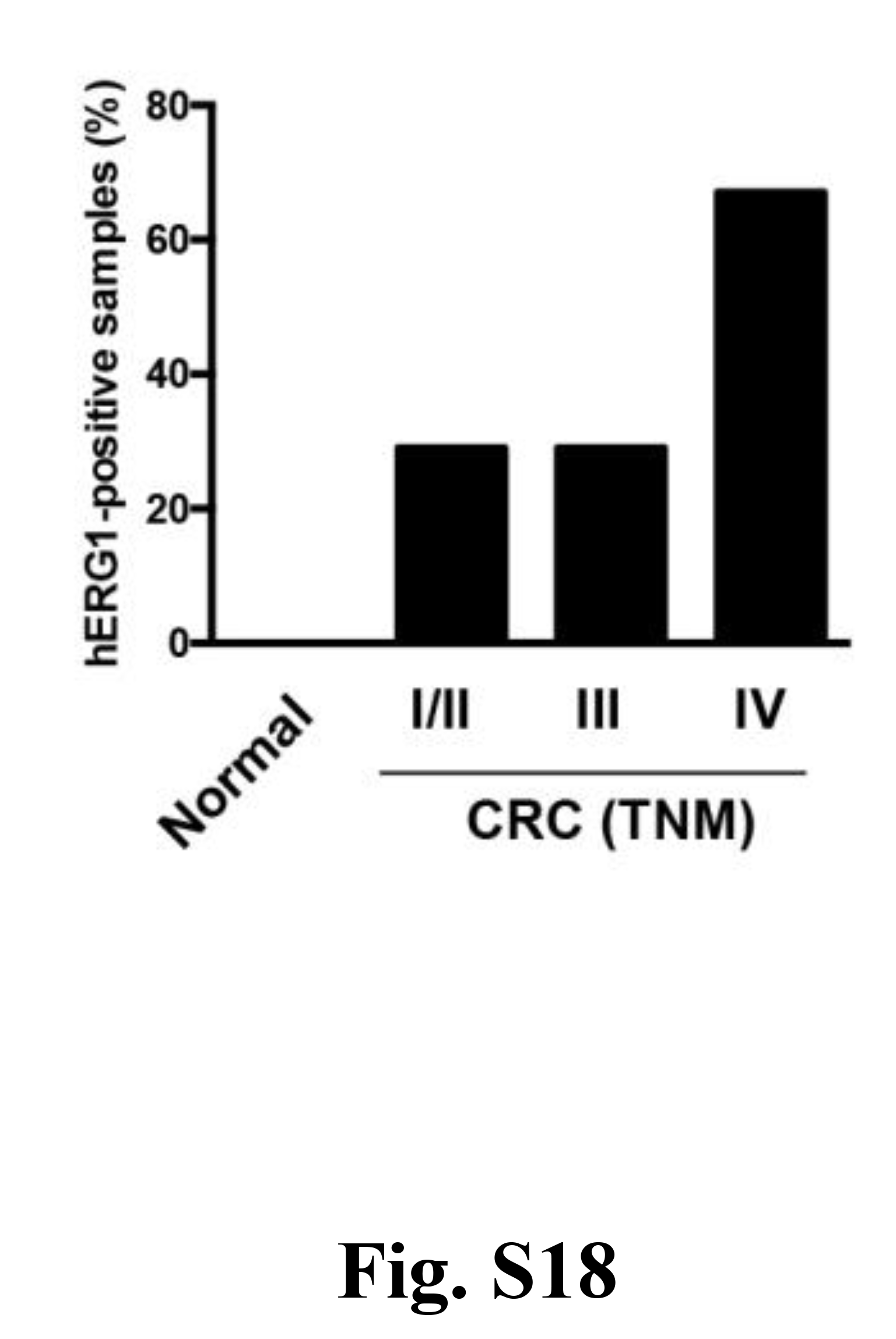

Supplement: Supplementary file 19 — Supplementary Figure S18 [file 41419_2020_2349_MOESM19_ESM.tif]
