# Supplementary material for: Clarithromycin inhibits autophagy in colorectal cancer by regulating the hERG1 potassium channel interaction with PI3K
Source: Cell Death Dis. 2020 Mar 2;11(3):161. doi: 10.1038/s41419-020-2349-8 (PMC7052256; doi:10.1038/s41419-020-2349-8)
Supplement: Supplementary file 20 — Supplementary Table S1 [file 41419_2020_2349_MOESM20_ESM.docx]

| **Table S1. List of antibodies used in the study.** | | | | |  |
| --- | --- | --- | --- | --- | --- |
| **Antibody Target** | **Antibody Name** | **Supplier (catalog #)** | **Type of Antibody** | **Dilution** | |
| Total Akt1, Akt2 and Akt3 proteins | AKT1/2/3 Antibody  (H-136) | Santa Cruz Biotechnology (#sc-8312) | Rabbit pAb | WB: 1:500 | |
| Akt phosphorylated at Thr^308^ | p-Akt1/2/3 Antibody  (Thr 308) | Santa Cruz Biotechnology (#sc-271966) | Mouse mAb | WB: 1:500 | |
| Full-length and large fragment of caspase-3 | Caspase 3 (8G10) Rabbit mAb | Cell Signaling Technology (#9665) | RAbbit mAb | WB: 1:1000 | |
| Total p44 and p42 MAP Kinase (Erk1 and Erk2) | ERK 1/2 Antibody (H-72) | Santa Cruz Biotechnology (#sc-292838) | Rabbit pAb | WB: 1:200 | |
| Erk1 and Erk2 phosphorylated at Thr^202^ and Tyr^204^ | Phospho-44/42 MAPK (Erk1/2) (Thr202/Tyr204) Antibody | Cell Signaling Technology (#9101) | Rabbit pAb | WB: 1:1000 | |
| hERG1 protein | hERG1 CT  pan–polyclonal antibody | Di.V.A.L. Toscana SRL (#DT-552) | Rabbit pAb | WB: 1:1000 | |
|  | anti-hERG1 monoclonal antibody | MCK Therapeutics | Rabbit mAb | IHC: 0,005 µg/µl final concentration  Co-IP: 5 µg antibody/mg protein | |
| Total LC3A and LC3B proteins | LC3A/B Antibody | Cell Signaling Technology (#4108) | Rabbit pAb | WB: 1:1000 | |
|  | Anti-LC3A/B antibody | Abcam  (#128025) | Rabbit pAb | IF: 1:200 | |
| Total LC3B protein | LC3B Antibody | Novus Biologicals  (#NB600-1384SS) | Rabbit pAb | IHC: 1:1000 | |
| p53 protein | P53 (DO-7) Monoclonal Antibody | DakoCytomation  (#M7001) | Mouse mAb | WB: 1:1000  IHC: 1:50 | |
| PI3K regulatory subunit alpha (p85) | Anti-PI3 kinase Antibody, p85 | Upstate  (#06-195) | Rabbit pAb | WB: 1:2000 | |
| SQSTM1/p62 protein | SQSTM1/p62 (D5E2) Rabbit mAb | Cell Signaling Technology (#8025) | Rabbit mAb | WB: 1:1000 | |
| Tubulin | Monoclonal Anti-α-Tubulin antibody produced in mouse | Sigma-Aldrich (#T9026) | mouse mAb | WB: 1:500 | |
| Secondary antibodies used for western blot: anti-rabbit IgG peroxidase-conjugated (Sigma-Aldrich #A0545; dilution 1:10000) and anti-mouse IgG peroxidase antibodies (Sigma-Aldrich #A4416; dilution 1:5000). | | | | | |
| Legend: Co-IP: co-immunoprecipitation; IF: Immunofluorescence; IHC: Immunohistochemistry; WB: western blot. | | | | | |
