# Supplementary material for: Clarithromycin inhibits autophagy in colorectal cancer by regulating the hERG1 potassium channel interaction with PI3K
Source: Cell Death Dis. 2020 Mar 2;11(3):161. doi: 10.1038/s41419-020-2349-8 (PMC7052256; doi:10.1038/s41419-020-2349-8)
Supplement: Supplementary file 21 — Supplementary Table S2 [file 41419_2020_2349_MOESM21_ESM.docx]

| **Table S2. List of chemicals used in the study.** | |  |
| --- | --- | --- |
| **Chemicals used for in vitro experiments** | | |
| **Chemical Name** | **Supplier (catalog #)** | **Solvent** |
| Clarithromycin | Sigma-Aldrich (#C9742) | DMSO |
| 5-Fluorouracil | Sigma-Aldrich (#F6627) | DMSO |
| Irinotecan | Sigma-Aldrich (#I1406) | DMSO |
| Bafilomycin A1 | Sigma-Aldrich (#19-148) | DMSO |
| Cisplatin | Sigma-Aldrich (#232120) | bi-distilled water |
| Oxaliplatin | Sigma-Aldrich (#O9512) | bi-distilled water |
| **Chemicals used for in vivo experiments** | | |
| **Chemical Name** | **Supplier** | **Dosage, schedule** |
| Macladin  (Clarithromycin 50 mg/ml) | Azienda Ospedaliera Universitaria Careggi (Florence, Italy) | 40 mg kg^-1^, twice daily by oral gavage |
| Fluorouracile Teva  (5-fluorouracil; 25 mg/ml) | Azienda Ospedaliera Universitaria Careggi (Florence, Italy) | 30 mg kg^-1^*, twice a week intraperitoneally |
| *30 mg kg^-1^, corresponds to the human equivalent dose (calculated as in Nair, A.B. & Jacob, S. A simple practice guide for dose conversion between animals and human. *J. Basic Clin Pharm* **7,** 27-31 (2016)), of 90 mg/m^2^, around one quarter of the drug regiment used for human CRC treatment (400 mg/m^2^) | | |
