# Supplementary material for: Clarithromycin inhibits autophagy in colorectal cancer by regulating the hERG1 potassium channel interaction with PI3K
Source: Cell Death Dis. 2020 Mar 2;11(3):161. doi: 10.1038/s41419-020-2349-8 (PMC7052256; doi:10.1038/s41419-020-2349-8)
Supplement: Supplementary file 22 — Supplementary Table S3 [file 41419_2020_2349_MOESM22_ESM.docx]

| **Table S3. Results of statistical analysis relative to Figs. 5A and 7A** | | | | |
| --- | --- | --- | --- | --- |
|  | **After 24 hours of treatment** | **After 48 hours of treatment** | **After 72 hours of**  **single treatment**  **(left panel)** | **After 72 hours of double treatment (right panel)** |
| **Fig. 4A** | ****P* < 0.001: Cla 80 vs Control, *P* < 0.05; Cla 160 vs Control, | ****P* < 0.001: Cla 40 vs Control; Cla 80 vs Control; Cla 160 vs Control. | ****P* < 0.001: Cla 40 vs Control; Cla 80 vs Control; Cla 160 vs Control. | ****P* < 0.001: Cla 40 vs Control, *P* < 0.01, Cla 80 and Cla 160 vs Control. |
| **Fig. 6A** | **P* < 0.05: 5-FU 2.2 + Cla 40 vs 5-FU 2.2; 5-FU 2.2 vs Control.  ***P* < 0.01: 5-FU 13.7 vs Control; Cla 80 vs Control; 5-FU 13.7 + Cla 80 vas Control; 5-FU 2.2 + Cla 40 vs Control*.* | **P* < 0.05: 5-FU 2.2 vs Control; 5-FU 13.7 vs Control; 5-FU 2.2 + Cla 40 vs Control; 5-FU 13.7 + Cla 80 vs 5-FU 13.7.  ***P* < 0.01: 5-FU 13.7 + Cla 80 vs 5-FU 13.7. | **P* < 0.05: Cla 80 vs Control.  ****P* < 0.001: 5-FU 2.2 vs Control; 5-FU 13.7 vs Control; 5-FU 2.2 + Cla 40 vs Control; 5-FU 13.7 + Cla 80 vs Control. | **P* < 0.05: Cla 40 vs Control; 5-FU 2.2 + Cla 40 vs 5-FU 2.2; 5-FU 2.2 + Cla 40 (double treatment) vs 5-FU 2.2 + Cla 40 (single treatment); 5-FU 13.7 + Cla 80 (double treatment) vs 5-FU 13.7 + Cla 80 (single treatment).  ****P* < 0.001: 5-FU 2.2 vs Control; 5-FU 13.7 vs Control; Cla 80 vs Control; 5-FU 2.2 + Cla 40 vs Control; 5-FU 13.7 + Cla 80 vs Control. |
